# Competitors alter selection on alpine plants exposed to experimental climate change

**DOI:** 10.1093/evlett/qrad066

**Published:** 2023-12-28

**Authors:** Hanna Nomoto, Simone Fior, Jake Alexander

**Affiliations:** Institute of Integrative Biology, Department of Environmental Systems Science, ETH Zürich, Zürich, Switzerland; Institute of Biology, Functional ecology laboratory, University of Neuchâtel, Neuchâtel, Switzerland; Institute of Integrative Biology, Department of Environmental Systems Science, ETH Zürich, Zürich, Switzerland; Institute of Integrative Biology, Department of Environmental Systems Science, ETH Zürich, Zürich, Switzerland

**Keywords:** alpine plants, aster models, competitive interactions, elevation gradient, global warming, phenotypic selection

## Abstract

Investigating how climate change alters selection regimes is a crucial step toward understanding the potential of populations to evolve in the face of changing conditions. Previous studies have mainly focused on understanding how changing climate directly influences selection, while the role of species’ interactions has received little attention. Here, we used a transplant experiment along an elevation gradient to estimate how climate warming and competitive interactions lead to shifts in directional phenotypic selection on morphology and phenology of four alpine plants. We found that warming generally imposed novel selection, with the largest shifts in regimes acting on specific leaf area and flowering time across species. Competitors instead weakened the selection acting on traits that was imposed directly by warming. Weakened or absent selection in the presence of competitors was largely associated with the suppression of absolute means and variation of fitness. Our results suggest that although climate change can impose strong selection, competitive interactions within communities might act to limit selection and thereby stymie evolutionary responses in alpine plants facing climate change.

## Introduction

Climate acts as a strong selective agent on natural populations (Etterson, 2004; Hoffmann & Sgrò, 2011; Jump & Peñuelas, 2005; Réale et al., 2003) and phenotypic responses to climate change are already reflected in changes in a number of traits, such as phenology (Lane et al., 2012; Menzel et al., 2006; Parmesan, 2006) and size (Björkman et al., 2018; Rammig et al., 2010; Van Buskirk et al., 2010), as well as in estimates of performance (Post & Forchhammer, 2008). Observed trait changes can reflect both plasticity and evolutionary responses to selection (Gienapp et al., 2008). While phenotypic plasticity may allow species to tolerate climate change in the short term (Ensing & Eckert, 2019), it is believed that plasticity may be insufficient to ensure long-term persistence when populations are confronted with novel environmental conditions (Sheth & Angert, 2014) and that adaptive responses will be essential to sustain fitness (Etterson, 2004; Lande & Shannon, 1996; Visser, 2008). Although observational studies can provide clear evidence for climate-associated shifts in phenotypes, it is necessary to measure climate-mediated selection, i.e., the effects of climate change on the relationship between phenotypic traits and fitness, to understand how climate change might influence trait evolution in natural populations. Studying how climate change affects selection regimes is an initial step toward understanding evolutionary responses and a key part of making predictions about future shifts of phenotypes in populations (Etterson, 2004; Liu & El-Kassaby, 2019).

Previous studies have mainly focused on understanding how alterations of abiotic factors directly influence selection regimes as climate changes (Anderson et al., 2012b; Jump et al., 2006). For example, studies of plants suggest that warmer and drier climates will favor thicker leaves (Etterson & Shaw, 2001), larger biomass (Sun et al., 2020), and earlier flowering phenology (Anderson et al., 2012b; Bemmels & Anderson, 2019; Franks et al., 2007). Warming can also select for smaller floral size as a consequence of reduced resource investment in floral structures under novel abiotic stress (Brunet & Etten, 2019; Galen, 2000; Yu et al., 2016). But in addition to directly influencing abiotic selection pressures, climate change is also causing shifts in biotic interactions, for example, reflected in alterations in the density (Elmendorf et al., 2012; Walker et al., 2006) and/or identity of competitors (Gilman et al., 2010).

Changing competitive interactions—which can be thought of as indirect effects of climate change—can have a large impact on species’ responses to future climatic conditions (Cahill et al., 2013; Tylianakis et al., 2008) and sometimes play an even larger role than direct effects of warming, for example, in reducing population growth of plants (Nomoto & Alexander, 2021). As competitors play a crucial role in shaping a plant’s environment, they are expected to generate changes in trait expression (Aschehoug et al., 2016; Bittebiere & Mony, 2014; Craine & Dybzinski, 2013) as well as plant performance (Callaway et al., 2011; Emery & Ackerly, 2014; Van Der Wal et al., 2000) via genotype × environment (G × E) interactions. Competition itself can therefore alter or impose new selection regimes on plant morphology and phenology (Barraclough, 2015; Calsbeek & Cox, 2010; Palacio-Lopez et al., 2020) and can, for example, drive evolution toward taller stature (Kooyers et al., 2017), increased specific leaf area (SLA; Bennett et al., 2016; Sheppard, 2019, however see Conti et al., 2018), and earlier flowering (Kooyers et al., 2017; Palacio-Lopez et al., 2020) as a consequence of limited light and/or resource availability.

Previous studies have mainly examined how climate change and competition independently influence selection regimes, but competition may occur in concert with selection imposed by abiotic agents (Lau et al., 2014; Lorts & Lasky, 2020; Osmond & de Mazancourt, 2013), with one factor acting to reinforce or constrain selection imposed by the other. For example, Lorts and Lasky (2020) showed that selection acted toward increased aboveground biomass for *Arabidopsis thaliana* only under well-watered conditions and in the presence of competitors, while selection was absent when competitors were removed and individuals experienced drought. However, the few previous studies that empirically estimate interactive effects of climate and competitors on selection have predominantly done so under controlled environments (Lau et al., 2014; Lorts & Lasky, 2020), which limit our understanding of the impact of climate change in natural populations. To understand how climate change alters selection imposed on natural populations, it is moreover necessary to identify the underlying processes driving shifts in selection (Box 1). Yet, how climate warming and changing competitive interactions affect the determinants of selection remains largely unknown.

Box 1. **Underlying processes generating differences in directional selection**

**Figure 1.**
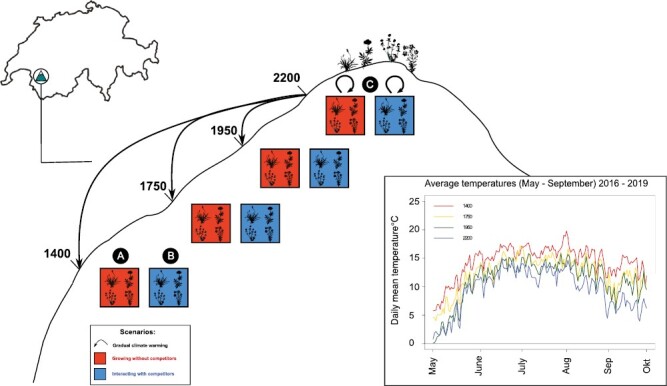
Transplant experiment design. Four alpine plant species were transplanted from 2,200 m to three lower elevation sites (1,950, 1,750, and 1,400 m) to simulate climate warming of, respectively, 1.65, 3.05, and 4.85 °C, representing the average growing season (May–September) temperatures during the experiment (2017–2019; inset). By planting individuals into bare soil we tested how direct effects of climate warming, in the absence of competitors, alter patterns of phenotypic selection (A). By planting individuals into vegetated plots (1 m^2^ turfs including rhizosphere soil to a depth of c. 20 cm; see *Methods* section), we tested how future competitive environments in combination with climate warming alter patterns of selection (B). Individuals were additionally transplanted into the 2,200 m site both with and without competitors to evaluate the performance of individuals in their current environment (C). Sample size at the start of the experiment was *n* = 100 for each competitor treatment (absence/presence of competitors) for each low-elevation site and *n* = 50 for each competitor treatment at the 2,200 m site. Note that soil and vegetation treatments were composed of local (low elevation) origins and 2,200 m origins that had been transplanted downward (see *Methods* section). For a detailed description of the experimental design see Supplementary Methods.

Differences in directional selection in response to changing environments could arise from shifts in each of the three key parameters used to estimate selection (Anderson et al., 2012a; Conner & Hartl, 2004; Futuyma & Kirkpatrick, 2018). First, a shift in the environment, such as warming or changing competitive interactions, may act to alter fitness associated with particular trait values, effectively changing the shape of the fitness function (Figure 1A). In this scenario, differences in directional selection are generated because different trait values become advantageous/disadvantageous following alterations in the environment. However, selection can change even if the shape of the underlying fitness function does not. On the one hand, the distribution of traits expressed in the population may change, so that trait values expressed by individuals growing in one environment (e.g., Figure 1, red) vs. another (e.g., Figure 1, blue) occupy different regions of the fitness function (Figure 1B). Such plastic responses may constrain selection if changes in the environment drive trait distributions far from their phenotypic optimum, where the fitness function is characterized by a weak or zero slope (Figure 1B, blue). On the other hand, selection regimes might be altered as a consequence of shifts in overall fitness (Figure 1C). In particular, the suppression of fitness of individuals at the tail of the distribution could alter the range of trait values effectively visible to selection (Figure 1C, where the dashed portion of the blue line indicates the absence of selection due to zero fitness values in the blue environment). Shifts in directional selection caused by changes in the shape of the fitness function (A) can be inferred when shifts in both trait (B) and fitness (C) distributions are nonsignificant. If trait and fitness distributions differ significantly, these processes can be invoked as potential drivers of altered selection regimes.

Figure 1. Alternative scenarios for how alterations in the shape of fitness functions (A), trait (B), and fitness distributions (C) can generate differences in directional selection. The plots illustrate hypothetical fitness functions (curves in upper panels) in two environments (red and blue), with trait values on the *x*-axis and fitness on the left-hand *y*-axis. Points illustrate fitness values corresponding to expressed trait values. Density plots illustrate trait distributions on the *x*-axis, with density shown on the right-hand *y*-axis. Mean trait values are indicated by vertical dashed lines. Lower panels illustrate inferences of directional selection. Note that these three scenarios are not mutually exclusive. Moreover, fitness functions are hypothetical, as empirical estimates can effectively be obtained only within the range of expressed trait variation.

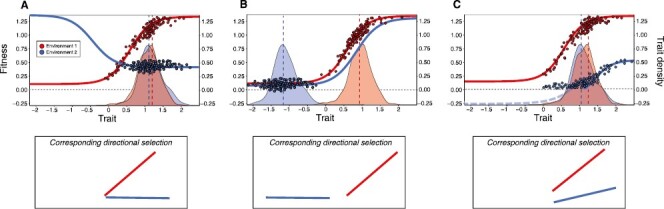

Understanding how alterations in both climate and competitive interactions shape selection regimes and identifying the underlying processes responsible for these potential shifts is particularly relevant for species facing climate change at high elevation, where climate warming is occurring at a faster rate than global averages (Pepin et al., 2015; Wang et al., 2016; You et al., 2020, but see Tudoroiu et al., 2016), and plants are experiencing shifts in the relative abundance and identity of neighboring species (Klanderud, 2005; Le Roux & McGeoch, 2008). While earlier studies have assessed the role of climate change in altering selection regimes acting on plants at high elevation (Anderson et al., 2012a; Bemmels & Anderson, 2019; Totland, 1999), the interactive effect of climate warming and competitive interactions in driving selection on high-elevation plants has, to our knowledge, never been tested. Here, we used a transplant experiment along an elevation gradient in the Swiss Alps to test whether climate change in combination with interactions with competitors lead to shifts in directional phenotypic selection acting on SLA, stalk height, floral size, and flowering time in four alpine plants. We asked: (a) Do climate change and competition, individually and in combination, influence phenotypic selection acting on traits of alpine plants? (b) Are selection pressures from climate change and competition aligned or antagonistic to each other?

We hypothesized that both climate warming and competition alter selection regimes on our focal species. More specifically, we expected warmer conditions to select for smaller SLA, typically favored under drought conditions (Hamann et al., 2017; Wellstein et al., 2017), and earlier flowering (Anderson et al., 2012a; Franks et al., 2007) as described earlier. Warmer conditions may also relax selection toward smaller stalk height, which is typically favored at high elevation (Halbritter et al., 2018; Körner, 2003). We hypothesized that competitors impose selection toward taller stalk height (Kooyers et al., 2017) and earlier flowering (Palacio-Lopez et al., 2020). In contrast, we expected competitors to favor larger SLA (Bennett et al., 2016; Sheppard, 2019), which may offset selection toward smaller SLA imposed by warming in scenarios where species experience warming in the presence of competitors. While selection on floral size is likely to be influenced by changing pollinator communities across the elevation gradient, we expected reduced resource allocation toward floral structures in response to both warming and competition to weaken selection toward increased floral size. In sum, we expected selection imposed by climate warming and competition to be aligned for flowering and floral size, but antagonistic for SLA.

## Materials and methods

### Study system

Our study was based on a transplant experiment conducted along an elevation gradient in the Swiss Alps (46˚13ʹ08N, 7˚03ʹ13E). The experiment included 4 sites situated at 2,200, 1,950, 1,750, and 1,400 m a.s.l., corresponding to a climate gradient where temperatures ranged from 9.5 °C to 14.3 °C throughout the experiment (May–September, 2016–2019; Figure 1). *Plantago alpina* (Plantaginaceae), *Anthyllis vulneraria* ssp. *alpestris* (Fabaceae, hereafter *A. alpestris*), *Trifolium badium* (Fabaceae), and *Campanula scheuchzeri* (Campanulaceae), which all are perennial forbs abundant above 2,000 m in the study area and widespread in the alpine zone of Switzerland (Atlas de la Flore Vaudois, 2022), were selected as focal species. For more information on species used in this study, see Supplementary Table S1.

### Experimental design

The experiment was designed to simulate scenarios in which alpine plants experienced incremental climate warming in the absence or presence of competitors. Climate warming was simulated by transplanting individuals of the focal species from 2,200 m to the three lower sites, corresponding to 1.65–4.85 °C warming of the mean growing season (May–September) temperature. While downhill transplantation along mountain gradients exposes plants to shifts in abiotic and biotic factors other than temperature, alterations of these factors are often congruent with expectations under climate change (see further discussion in Supplementary Methods). To simulate scenarios in which alpine plants experience direct effects of climate change in the absence of competitors (Figure 1A), we planted individuals into bare soil at each lower elevation site. We also simulated warming in the presence of competitors (Figure 1B) by planting individuals into 1 m^2^ turfs of intact plant communities including rhizosphere soil (to a depth of c. 20 cm) at each lower elevation site. As a “no warming” control, individuals were replanted at the 2,200 m site both in the absence (bare soil) and presence (intact plant communities) of competitors (Figure 1C).

In the original design of the experiment, individuals growing in the presence of competitors were planted either into turfs originating from the 2,200 m site that had been transplanted down to each of the four sites (*n* = 10 turfs per site) or into local turfs excavated and replanted at each of the three lower elevation sites (1,400, 1,750, and 1,950 m; *n* = 10 turfs per site; Supplementary Figure S1). Likewise, bare soil treatments (absence of competitors) were represented by two different soil origins, where focal species grew on soil originating from either high or local sites at low elevation (*n* = 10 bare soil plots per site and soil origin). While the origin of competitors/soil origin may impact selection regimes, low sample size within each competitor/soil treatment prevented us from reliably estimating selection from each competitor/soil treatment. Instead, to maximize sample size for estimates of selection, we pooled the two competitor or two soil treatments within each site to test the effects of warming and competition per se on phenotypic selection. While competitor/soil origin treatments therefore were not the focus of the current analyses, we nonetheless investigated their potential effect on selection estimates by testing differences in fitness/traits between competitor/soil origins (Supplementary Figures S2 and S3, Supplementary Tables S2–S5). For more information on transplant design, differences in communities between sites and pooling across competitor/soil origins, see Supplementary Methods, Supplementary Figure S4, and Nomoto and Alexander (2021). At each site, each turf/soil treatment was randomly allocated to four positions (1 m^2^ plots) within 10 blocks. In total, across the whole experiment, individuals were planted into 140 plots (20- low and 20 high-elevation turfs/soil plots at low-elevation sites and 20 high-elevation turfs/soil plots at the highest site; Supplementary Figure S1). Sites were fenced to avoid grazing animals, but all plants within turfs were clipped to c. 15 cm at the end of growing seasons to simulate grazing.

Focal species were collected from around the 2,200 m site (sampling area of c. 0.27 km^2^) by excavating single ramets of adult individuals (hereafter “individuals”). At the end of September 2016, individuals were planted (13 cm apart) at randomly selected positions within a regular grid in each plot (five individuals/species/plot; i.e., *n* = 100 per competitor treatment (competitors present vs. absent, i.e., bare soil) for each low-elevation site and *n* = 50 per treatment for the high-elevation site with total *N* = 2,800 individuals at the start of the experiment). By the end of the growing season 2017, dead individuals of *A. alpestris* (*n* = 282), *T. badium* (*n* = 421), and *P. alpina* (*n* = 100) were replaced. For a detailed description of the experimental design see Supplementary Methods.

### Assessment of traits and fitness

In each of 3 years (2017–2019), we estimated stalk height (height of the tallest stalk), SLA, and leaf number toward the end of the growing season (late August). SLA was measured on the largest undamaged leaf per individual. Floral size was measured as the banner length for *A. alpestris* and surface area of the corolla for *C. scheuchzeri*; flowers of *P. alpina* and *T. badium* are very small and could not be measured accurately in the field, so this trait was not recorded for these species. We recorded the timing of flowering (Julian day) weekly throughout the growing season (May–September). As soon as sites were accessible (May–June) we scored survival. By the end of the growing season (September), seeds were collected and the number of intact seeds were counted to estimate the total number of seeds produced per individual. For details of assessments of traits and seed production see Supplementary Methods.

### Estimating fitness with aster models

We applied aster models (Geyer et al., 2007), integrating viability (i.e., survival), and fecundity (i.e., flowering probability and seed production) for the duration of the experiment to generate a combined estimate of individual fitness (Supplementary Figure S5). By accounting for the contributions of multiple life history traits, aster models are appropriate for predictions of lifetime fitness (Geyer et al., 2007). We fitted aster models using the R package “aster,” in which response variables are represented by a vector of fitness components modeled by its appropriate statistical distribution (survival and flowering probability were modeled as Bernoulli and seed number as Poisson). Individuals that were planted into the experiment in 2017 (replacing individuals that died the first year) were excluded from the first year in aster models. We constructed models for each trait, warming level and competition treatment separately to predict fitness across traits and warming/competitor treatments. Traits as well as leaf number, which is the trait most strongly correlated with biomass for the species included in this study (Nomoto & Alexander, 2021), were included as fixed factor in the aster models. By including leaf number in the models, we account for expected effects of size on fitness estimates. As we aimed to quantify selection acting throughout the whole period of the experiment, we implemented averaged trait values (and leaf number) throughout the experiment (2017–2019) as explanatory variables in aster models. Traits were significantly correlated across years for most species except stalk height and floral size for *A. alpestris*, stalk height for *T. badium*, and floral size and flowering time for *C. scheuchzeri* (Supplementary Table S6). For consistency between models used to estimate fitness and selection, traits and leaf number were standardized (mean = 0 and *SD* = 1) across the experiment before fitting the aster models.

### Quantifying the effect of warming and competition on selection differentials

We quantified selection using regression analyses describing the relationship between a trait and fitness (Lande & Arnold, 1983). Individual fitness predicted by aster models was first relativized (e.g., Ensing et al., 2021; Peschel et al., 2021; Souto-Vilarós et al., 2018) by dividing individual fitness estimates from the aster models by the overall mean fitness across warming and competitor treatments. Relative fitness was then implemented as the response variable in the fitness functions. Both warming (represented by the experimental sites; Figure 1) and competition were implemented as fixed environmental treatments as we aimed to preserve any differences in the distribution of traits and fitness between these treatments when estimating their effect on selection. Therefore, we standardized traits and relativized fitness globally across the experiment (De Lisle & Svensson, 2017).

To select minimum models from which to estimate phenotypic selection, we first determined whether warming and competition had significant effects on the relationship between relative fitness and each standardized trait. To disentangle phenotypic selection acting on traits from the simple relationship between fitness and size, we additionally included standardized leaf number as a fixed factor in the models. We used linear models for which fitness was cube root transformed before relativization and SLA was log transformed to obtain normally distributed residuals. We selected the most parsimonious model based on likelihood ratio tests comparing full and reduced models.

The structure of the most parsimonious models identified by the model selection procedure was used to estimate selection differentials using linear models. Terms from the most parsimonious model were implemented as fixed effects in models. For *P. alpina* and *A. alpestris* we fitted linear mixed effects models, since experimental block within each site (see Supplementary Figure S1) could be implemented as a random effect without convergence issues. To estimate the significance of selection differentials and compare estimates between treatments, we performed parametric bootstraps (*n* = 5,000) to obtain replicates of the model coefficients and calculated mean selection differentials and their 95% confidence intervals. We also determined whether selection differed between climate change scenarios and when growing in the absence/presence of competitors by calculating the differences between each bootstrap replicate pair (*n* = 5,000) for treatment comparisons of interest. We estimated 95% confidence intervals of these differences to determine whether selection gradients were significantly different between treatments (Barbour et al., 2020). For a detailed description of how selection differentials were estimated, see Supplementary Methods.

### Differences in absolute means and variation of fitness and traits

We performed Tukey’s Honest Significant Difference and analyses of heterogeneous variance to examine pairwise differences in the mean and variation, respectively, of phenotypic traits and fitness between warming and/or competitor treatments. False discovery rate corrections were applied to adjust *p*-values obtained from multiple comparisons of means within traits and species. Correlations between traits across treatments were estimated with Pearson’s correlation coefficient. To explore the underlying mechanisms generating shifts in selection regimes across treatments, we illustrated the relationship between relative fitness and standardized traits in combination with density plots showing trait distributions. Analyses were performed in R version 4.2.1 (R Core Team, 2021).

## Results

### Anthyllis alpestris

**Figure 2.**
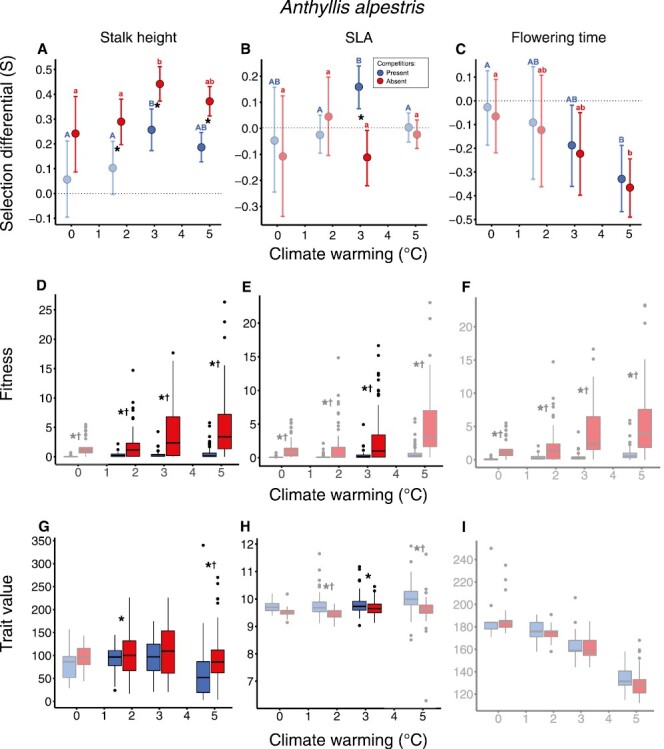
Selection differentials (A–C), the distribution of fitness (D–F), and phenotypic traits (G–I) across factors (warming and competition) for which selection was estimated for *A. alpestris.* The effect of site is plotted as the level of warming on the *x*-axis, simulated by transplantation to lower elevations (Figure 1). (A)–(C) show estimates and 95% confidence interval from bootstrapped selection differentials (*n* = 5,000) where nonsignificant selection differentials are shown as partly transparent. Letters in (A)–(C) indicate significant differences between warming levels when competitors are present (capital letters) or when competitors are absent (lowercase letters), while asterisks indicate significant differences between competitor treatments. Significant differences in mean and variation of fitness (D–F) and traits (G–I) between competitor treatments are indicated by crosses and asterisks, respectively. In these plots (D–I), cases for which shifts in selection in response to competitors are not significant are shown as partly transparent. Note that significant differences in fitness (D–F) and fitness (G–I) variation/means only are indicated for competitor treatments here, while significant differences in selection differentials, fitness, and trait values across both warming and competitor treatments can be found in Supplementary Figure S10 and Supplementary Tables S8 and S12. Selection differentials are illustrated as slopes (fitness on *y*-axis and traits on *x*-axis) in combination with density plots illustrating trait distributions in Supplementary Figure S6.

Selection favored taller stalks under all levels of warming and smaller SLA under intermediate levels of warming for *A. alpestris* (Figure 2A and B; Supplementary Figure S6A and B). Selection on flowering time shifted from being nonsignificant under no warming to negative (i.e., toward earlier flowering) under high levels of warming (Figure 2C; Supplementary Figure S6C). Competitors acted to eliminate selection under low levels of warming while weakening selection toward taller stalk height under moderate and high levels of warming. Competitors also shifted selection from smaller to larger SLA under intermediate levels of warming. Weakened and nonsignificant selection toward taller stalk height in the presence of competitors was associated with substantial reductions in the mean and variation of fitness across all warming levels, in addition to reduced trait variation under low and high levels of warming (Figure 2D and G; Supplementary Figure S6A, Supplementary Table S12).

### Trifolium badium

**Figure 3.**
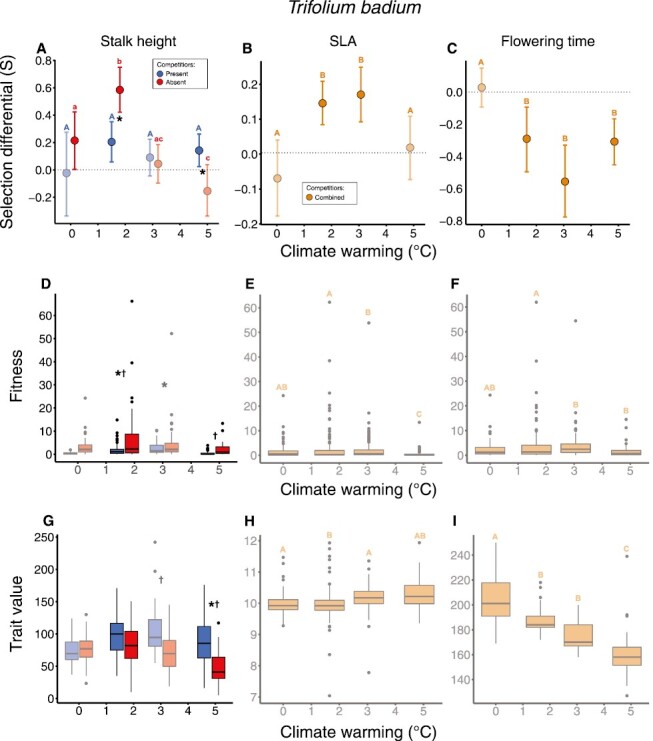
Selection differentials (A–C), the distribution of fitness (D–F), and phenotypic traits (G–I) across factors (warming and competition) for which selection was estimated for *T. badium.* See figure text of Figure 2. Significant differences in fitness and trait values across both warming and competitor treatments can be found in Supplementary Figure S11 and Supplementary Tables S9 and S13. Selection differentials are illustrated as slopes (fitness on *y*-axis and traits on *x*-axis) in combination with density plots illustrating trait distributions in Supplementary Figure S7.

Warming altered selection on stalk height for *T. badium*, in that selection acted toward taller stalks under the no warming control and low levels of warming, while selection differentials were nonsignificant under higher levels of warming (Figure 3A; Supplementary Figure S7A, Table 1). High levels of warming eliminated the selection toward larger SLA that was observed under lower levels of warming (Figure 3B; Supplementary Figure S7B). Selection acting on flowering time shifted significantly from being absent under no warming control to negative (i.e., toward earlier flowering) under all levels of warming (Figure 3C; Supplementary Figure S7C). Selection on stalk height was absent under no warming controls in the presence of competitors, while competitors weakened selection toward taller stalk heights under low levels of warming. Under high levels of warming, selection on stalk height shifted from nonsignificant in the absence of competitors to positive in the presence of competitors. Shifts from positive to negative selection acting on SLA following an increase from lower to high levels of warming were associated with increased variation and means of fitness (Supplementary Figures S7B and S11E, Supplementary Table S13). The shift from nonsignificant to positive selection on stalk height in the presence of competitors under high levels of warming was associated with increased trait variation and mean (Figure 3D; Supplementary Figure S7B, Supplementary Table S13). Weakened selection toward taller stalk heights under low levels of warming in the presence of competitors was associated with reductions in mean and variation in fitness.

**Table 1.** Models of focal species fitness as a function of trait, site, competition and their interactions.

Species	Trait name	Most parsimonious model	*N*
*A. alpestris*	Stalk height	Trait × Site (0.012) + Trait × Competition (<0.001) + Site × Competition (0.023)	420
	SLA	Trait × Site × Competition (0.005)	605
	Flowering time	Trait × Site (0.011) + Trait × Competition (0.040)	379
	Floral size	Site (<0.001) + Competition (<0.001) + Trait (0.032)	347
*T. badium*	Stalk height	Trait × Site × Competition (<0.001) ^*^	314
	SLA	Trait × Site (0.001) + Site × Competition (<0.001) ^*^	497
	Flowering time	Trait × Site (<0.001) + Site × Competition (<0.004)^*^	302
*P. alpina*	Stalk height	Trait × Site × Competition (0.016)	453
	SLA	Trait × Site (0.01) + Site × Competition (<0.001)	682
	Flowering time	Site × Competition (0.001) + Trait (<0.001)	438
*C. scheuchzeri*	Stalk height	Trait × Site × Competition (0.047)^*^	194
	SLA	Trait × Site × Competition (0.009)^*^	332
	Flowering time	Trait × Site × Competition (0.049)^*^	167
	Floral size	Trait × Competition (0.006) + Site (0.004) ^*^	128

*p*-values from *F*-tests for each model term are shown in parenthesis for most parsimonious models. Most parsimonious models were identified by comparisons between more complex and reduced models, including an intercept-only null model (“null”; see Supplementary Methods). If interactions were significant, *p*-values for main effects were not calculated. Effects were considered significant at *p* < .05. *N* indicates sample size for each species and trait. For effects of size see Supplementary Table S7.

^*^Random “Block” factor (see *Materials and methods*) was excluded from models due to convergence issues.

### Plantago alpina

**Figure 4.**
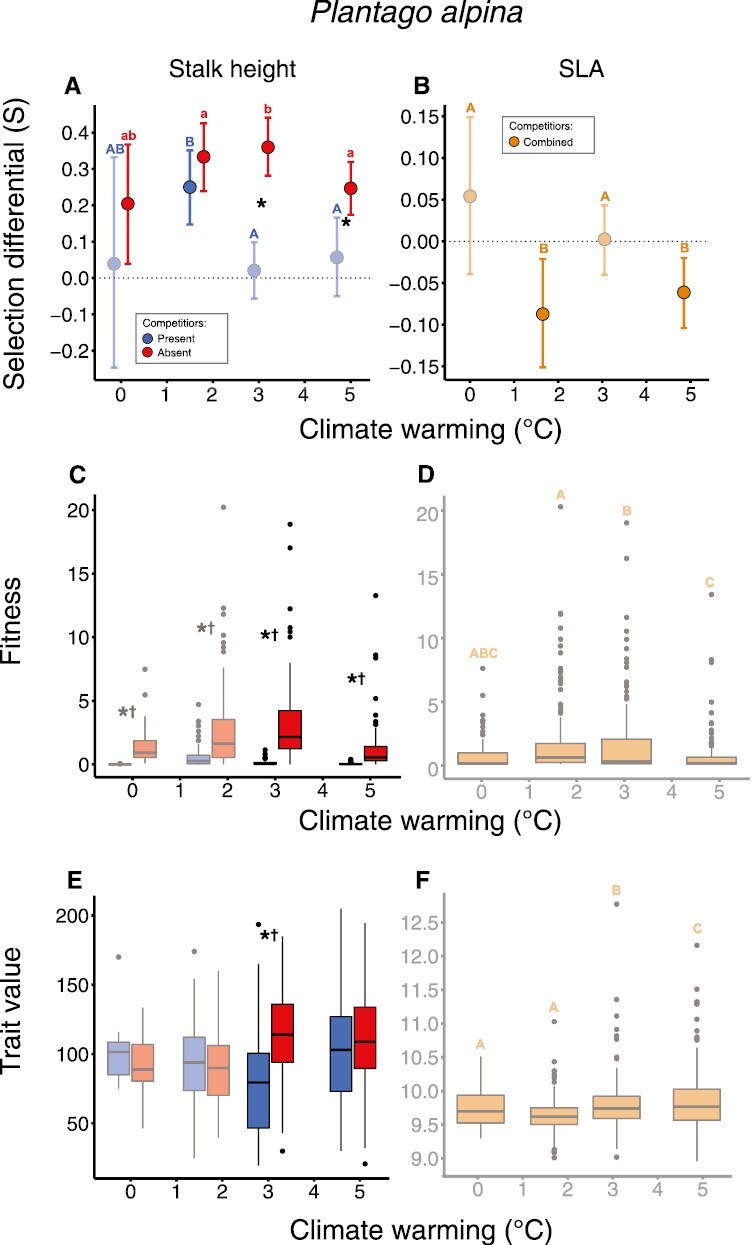
Selection differentials (A and B), the distribution of fitness (C and D), and phenotypic traits (E and F) across factors (warming and competition) for which selection was estimated for *P. alpina.* See figure text of Figure 2. Significant differences in fitness and trait values across both warming and competitor treatments can be found in Supplementary Figure S12 and Supplementary Tables S10 and S14. Selection differentials are illustrated as slopes (fitness on *y*-axis and traits on *x*-axis) in combination with density plots illustrating trait distributions in Supplementary Figure S8.

Selection toward taller stalks was observed across all warming levels for *P. alpina* and was stronger under moderate compared to high levels of warming (Figure 4A; Supplementary Figure S8A). Low and high levels of warming also imposed selection toward smaller SLA (Figure 4B; Supplementary Figure S8B). Competitors acted to eliminate selection toward taller stalks under moderate and high levels of warming. These shifts were associated with suppressed means and variation in fitness, while differences in trait values were significant under moderate levels of warming (Figure 4C and E; Supplementary Figure S8A, Supplementary Table S14).

### Campanula scheuchzeri

**Figure 5.**
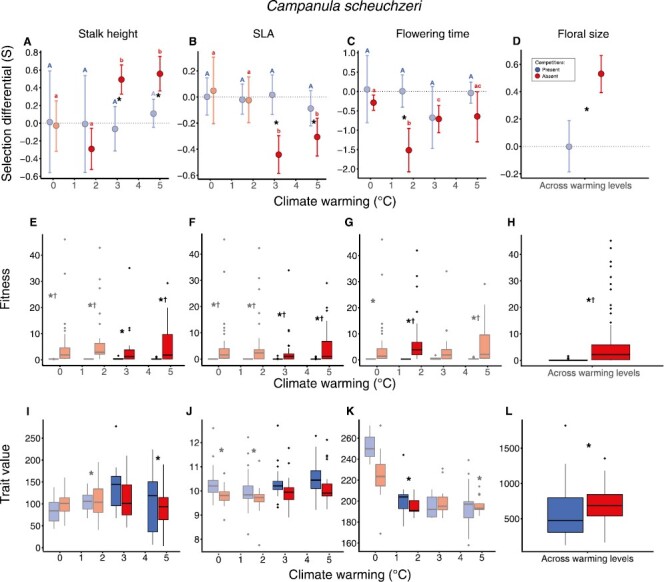
Selection differentials (A–D), the distribution of fitness (E–H), and phenotypic traits (I–L) across factors (warming and competition) for which selection was estimated for *C. scheuchzeri.* See figure text of Figure 2. Significant differences in fitness and trait values across both warming and competitor treatments can be found in Supplementary Figure S13 and Supplementary Tables S11 and S15. Selection differentials are illustrated as slopes (fitness on *y*-axis and traits on *x*-axis) in combination with density plots illustrating trait distributions in Supplementary Figure S9.

For *C. scheuchzeri*, moderate and high levels of warming imposed selection toward taller stalks, while selection was absent under no warming controls (Figure 5A; Supplementary Figure S9A). Smaller SLA was favored under moderate and high levels of warming, while selection was absent under no warming controls and low levels of warming (Figure 5B; Supplementary Figure S9B). Selection toward earlier flowering was observed under all warming levels but was stronger under moderate levels of warming compared to no warming controls (Figure 5C; Supplementary Figure S9C, Table 1). Larger floral size was favored independent of warming level (Figure 5D; Supplementary Figure S9D). Competitors eliminated significant selection observed for SLA and floral size in addition to selection under moderate and high levels of warming for stalk height. The absence of selection under competition was associated with reductions in both fitness means and variation for all traits (Figure 5E–H, Supplementary Table S15), while trait variation tended to be higher in the presence of competitors (Figure 5I–L; Supplementary Figure S9, Supplementary Table S15).

## Discussion

### Competitors limit selection imposed under warming

Assessing how climate-driven shifts in selection regimes act on natural populations is an initial step to understanding their ability to evolve in response to climate change. We found that plants transplanted to lower, warmer, elevations mainly experienced novel selection, for example, toward smaller SLA for *P. alpina* and *C. scheuchzeri*, earlier flowering time for *A. alpestris* and *T. badium*, as well as taller stalk height for *C. scheuchzeri*. In other cases, warming intensified selection, demonstrated by the strengthened selection toward taller stalk height for *A. alpestris* and *T. badium* and earlier flowering for *C. scheuchzeri*. Although previous studies suggest that climate change generally acts to strengthen selection (Anderson et al., 2012a; Bemmels & Anderson, 2019; Sun et al., 2020), we also found that warming may weaken selection in some cases, for example, for stalk height in *T. badium* under high levels of warming.

In addition to climate warming, competitive interactions played an important role in shaping selection regimes. Previous studies have shown that increased competition for light drives selection toward taller stature (Kooyers et al., 2017) and increased SLA (Bennett et al., 2016; Sheppard, 2019). We found some results consistent with these studies, since competitors imposed selection toward taller stalk height under high levels of warming for *T. badium* and toward larger SLA under moderate levels of warming for *A. alpestris*. However, competitors mainly acted to weaken or eliminate selection on traits, especially under warmer climates. This was clearly demonstrated by weakened selection toward taller stalk height for all species and toward smaller SLA and larger floral size for *C. scheuchzeri* in the presence of competitors. These results suggest that although climate warming can introduce novel selection regimes, the presence of competitors might act to reduce selection imposed directly by climate. Indeed, these effects of competition on selection might even be intensified under changing climate, which might occur due to an increased density of competitive species, such as graminoids (Klanderud, 2005), or the upslope migration and establishment of taller species from low elevation (Björkman et al., 2018) might act to reduce selection imposed by direct effects of warming itself.

Competitors might influence selection regimes via several, nonmutually exclusive pathways (Box 1). While we cannot exclude the possibility that the presence of competitors effectively causes changes in the fitness function (Box 1; Figure 1A), we did not identify any cases in which shifts in the fitness function could be isolated from associated changes in the distributions of trait and fitness values. Trait plasticity (Box 1; Figure 1B) in general also had little impact on the effect of competition on selection regimes, as we frequently observed overlapping trait distributions when shifts in selection regimes were observed across competitor treatments. However, plasticity may have played a role in altering selection regimes in some cases. For example, weakened selection toward taller stalk height in the presence of competitors under moderate levels of warming was associated with the reduction of absolute means of stalk height of *P. alpina* in the presence of competitors. This shift toward smaller stalk height in response to competition may have limited selection by driving the trait distribution away from the optimum (as illustrated in Box 1; Figure 1B, blue). However, the trait shift in this case occurred concomitantly with changes in fitness parameters, therefore, both processes likely acted jointly to generate the altered selection regime.

Our results strongly suggest that changes in fitness play a major role in altering selection regimes in response to competition (Box 1; Figure 1C). Weakened selection in the presence of competitors was frequently associated with declines in the mean and especially the variation of fitness. This was the case for selection acting toward taller stalk height for all species, in addition to smaller SLA and larger floral size for *C. scheuchzeri*. Similarly, previous studies have noted how intensified competition can suppress fitness (e.g., Iler & Goodell, 2010; Stanton-Geddes et al., 2012) and therefore also the strength of selection (Emery & Ackerly, 2014). This could reflect fitness trade-offs in environments where the availability of resources is limiting (Hamann et al., 2021; Reznick, 1985), such as under intense competition. Reduced availability of light, water, and nutrients in the presence of competitors may result in compromised reproductive output when resource allocation to vegetative growth and survival rather than seed production is favored (Gadgil & Solbrig, 1972; Lyu & Alexander, 2023; Tracey & Aarssen, 2014).

While our results suggest that reduction in fitness is a key driver of altered selection regimes under competition, in some cases shifts in selection under warming seemed to be driven by shifts in the trait values that were advantageous, i.e., changes in the fitness function (Figure 1A). For example, the shift from negative to positive selection on stalk height when warming increased from low to high levels of warming for *C. scheuchzeri* was not associated with significant changes in either traits or fitness (Supplementary Table S15) and may reflect the benefit of producing taller stalks to increase pollinator success (Aspi et al., 2003; Walsh et al., 2014) when selection toward shorter statures favored at higher elevations is relaxed (Körner, 2003). These hypotheses would need to be tested by performing additional experiments, such as supplemental hand pollination (e.g., Benoit & Caruso, 2021; Trunschke et al., 2020).

### Warming advances flowering time

In line with previous observations of high-elevation plants (Arft et al., 1999; Dorji et al., 2020; Høye et al., 2007; Jabis et al., 2020), flowering time advanced under warming for *A. alpestris*, *T. badium*, and *C. scheuchzeri*. The earlier initiation and prolonged period of climatic conditions beneficial for growth at lower elevations no doubt allowed individuals to flower earlier. But in addition, warming strengthened selection toward earlier flowering. Similarly, a study by Anderson et al. (2012a), combining long-term observational data with field experiments, suggested that earlier flowering in subalpine populations of *Boechera stricta* was driven by directional selection imposed by climate change. While early flowering at high elevations risks frost damage (Kudo, 2021) and unsuccessful pollination (Kudo & Cooper, 2019) risks are significantly reduced at lower elevations where favorable temperatures and pollinators are available during a longer period. Instead, early flowering is likely to be selected under warmer climates as it allows individuals to maximize fitness throughout the growing season (Arft et al., 1999; Henry & Molau, 1997). While we cannot identify the underlying processes driving selection toward earlier flowering under warming, our results indicate that flowering time is a key trait through which climate warming can shape both ecological and evolutionary responses in alpine plants.

### Caveats and future perspectives

Our study shows that although climate change can act as a strong selective agent on both morphology and phenology of alpine plants, competitive interactions can act to suppress novel selection regimes imposed directly by climate warming. Due to limited sample size, estimates of selection accounting for nonlinear terms, trait correlations (Supplementary Figure S14), and temporal fluctuations in selection across years (Supplementary Methods, Supplementary Table S16) could not be obtained. Although we are unable to tease apart direct selection on target traits underlying adaptive evolution, our comparative estimates strongly suggest that changes in evolutionary trajectories in response to competition are linked to population fitness. However, it is important to note that apart from survival and reproduction, other fitness proxies that were not included in this study, such as germination and recruitment rates, could impact observed estimates of lifetime fitness and therefore also selection. Importantly, evolution in response to novel selection regimes requires that traits under selection are heritable (Conner & Hartl, 2004; Falconer, 1996). However, achieving empirical estimates of heritability in the wild is challenging. As heritability is not a fixed property of a trait but varies with the environment, it is likely to differ between experimental treatments. Realistic estimates of shifts in heritability in response to changing environments thus require large sample sizes, which are often hard to obtain in experimental studies performed in the field (Ramakers et al., 2018). In this study, we estimated the heritability of traits for one of the species, *P. alpina*, by removing environmental effects simulated across the experiment (Supplementary Methods, Table S17). In line with previous studies performed in the field (Castellanos et al., 2015; Sedlacek et al., 2016; Wilson et al., 2006), trait heritability was low and varied substantially over years (Supplementary Table S18). We found that SLA was heritable, suggesting that this trait shows greater potential to respond to selection compared to other traits assessed for *P. alpina*. Although challenging, studies assessing the impact of climate warming and competitive interactions on the heritability of traits of natural populations are necessary to understand the ability of alpine plants to evolve in response to novel selection regimes.

Our experimental design simulates extreme scenarios, where alpine plants face rapid changes in climate and competitive communities. In reality, plants are more likely to face gradual warming and a temporally dynamic community including unknown densities and compositions of competitors (Alexander et al., 2018; Block et al., 2022). While we could not reliably estimate whether competitor effects on selection varied depending on the origin of competitors or soils (i.e., current high- or novel low-elevation species/alpine or low-elevation soil), we observed suppression of the mean and variation in fitness by both current and novel species (Supplementary Figures S2 and S3, Supplementary Tables S2–S5), indicating that the suppression of selection under competition may be, at least partly, independent of competitor or soil origin. Assessing how alterations in other abiotic and biotic variables, such as competitor origin, shape selection regimes is important targets for future work to better understand species’ responses to climate change.

In sum, our study suggests that competitive interactions may limit the potential of some alpine plants to evolve in response to selection imposed by climate warming, by suppressing fitness variation, and therefore reducing the evolutionary potential of traits. These results highlight the importance of estimating possible evolutionary consequences of climate change under realistic future scenarios by accounting for both novel climatic conditions and alterations in species interactions, as both these processes act jointly to shape future selection regimes.

## Supplementary Material

qrad066_suppl_Supplementary_Tables_S1-S15_Figures_S1-S15

## Data Availability

Data and code are deposited in the Dryad Digital Repository: https://doi.org/10.5061/dryad.v6wwpzh30
