# Supplementary material for: Competitors alter selection on alpine plants exposed to experimental climate change
Source: Evol Lett. 2023 Dec 28;8(1):114–27. doi: 10.1093/evlett/qrad066 (PMC10871967; doi:10.1093/evlett/qrad066)
Supplement: qrad066_suppl_Supplementary_Tables_S1-S15_Figures_S1-S15 [file qrad066_suppl_supplementary_tables_s1-s15_figures_s1-s15.pdf]

Supporting Information for Nomoto, Fior and Alexander (2024). Competitors alter selection on alpine plants exposed to experimental climate change. *Evolution Letters*.

SUPPLEMENTARY METHODS

Experimental design

Simulations of climate warming and altered soil/community origins

The competitor treatment simulated in the experiment (Fig. 1B) consisted of two distinct competitor origins: high elevation species that alpine plants currently interact with at high elevation (2220 m), representing “current competitors”; and species from lower elevations that are expected to migrate upwards as temperatures rise, representing “novel competitors”. Likewise, bare-soil treatments (absence of competitors; Fig. 1A) consisted of soil originating from either high or low elevation. The competitor/soil origin treatments were used to test the effects of competitor/soil origin on population dynamics of alpine plants facing warming in a previous study (Nomoto and Alexander 2021), yet not the focus of this study. For differences in absolute means and variation of fitness and traits across competitor/soil origins see Fig. S2-3, Table S2-5. For differences in plant community composition across sites see Fig. S4.

Variation in other abiotic and biotic factors across elevational gradients

Other factors apart from temperature, such as precipitation and UV radiation, also vary with elevation. Precipitation commonly declines with decreasing elevation (Rowe *et al.* 2008; Van Beusekom *et al.* 2015) and is thus consistent with projections

for future alterations in summer precipitation in this region (National Centre for Climate Services, Switzerland), while changes in UV radiation are considered to have negligible impacts on alpine plant growth (Körner 2003). Biotic factors, such as the identity and density of pollinators, also vary with elevation (Chesshire *et al.* 2021). Nonetheless, the downward transplantation performed here is expected to have caused shifts in pollinators and herbivores in a direction congruent with expectations of future alterations of these factors as climate changes. As insects migrate towards higher altitudes as climate warms (Parmesan 2006; Chen *et al.* 2011), the downhill transplantation of the focal species exposes them to similar communities of insect pollinators and herbivores that they are expected to encounter in the future (Richman *et al.* 2020). Potential shifts in pollinator communities and their interactions with plants along elevation were, however, not obtained in this study.

Measurements of traits and seed production

Stalk height

Stalk height was estimated as the stretch height from ground level to the highest reproductive structure (flower head for *P. alpina*, *A. alpestris, T. badium* and flower for *C. scheuchzeri*).

SLA (Specific leaf area)

SLA was estimated by dividing the area of the largest, undamaged fresh leaf (estimated with a CanoScan LiDE 300) by its dry weight (to the nearest 0.1 mg). Leaf area was estimated immediately after collection, by the end of the growing season

(late August), while dry weight was estimated later in the year of collection. To obtain a normal distribution (Lande and Arnold 1983), SLA was log-transformed before standardization.

Banner length/floral size

Floral size was estimated as banner length for *A. alpestris* and the surface area of the corolla based on measurements of the height (*h*) and the radius (*r*) of the corolla opening part for *C. scheuchzeri* ($\pi r^2 + \pi rh$). For *T. badium* and *P. alpina* estimates of floral size were hard to measure in field and we instead only estimated the size of the flower head directly. Flower head size reflects the potential of producing seeds (i.e. one of the fitness proxies used in this study), which complicates the interpretation of estimates of phenotypic selection on acting on flower head size. Therefore, selection acting on floral size was not estimated for these species. Floral size was measured when the majority of flowers on a given individual were fully developed and estimated by averaging across three flowers per individual.

Flowering time

Flowering time was estimated as the first day when an open flower was recorded (Julian day) and recorded weekly from May (or as shortly after snowmelt for sites at higher elevations as access permitted) until the end of the field season (September-October).

Seed production

Seed number was estimated by counting the number of mature and undamaged seeds per individual collected from all flowers (for *C. scheuchzeri*) or flower heads

(for *P. alpina*, *A. alpestris* and *T. badium*), when possible. For individuals producing multiple inflorescences, we collected seeds from 1-3 flowers (for *C. scheuchzeri*) or flower heads (*P. alpina*, A*. alpestris* and *T. badium*) and estimated total seed production by multiplying the average number of seeds produced per flower/flower head by the total number of flowers/flower heads produced by the individual.

Statistical analyses

<u>Selecting the most parsimonious models</u>

To identify the most parsimonious models, we fitted full models with relativized fitness as response variables and standardized trait, site (simulating climate change; Fig. 1), competition (absence/presence) and the two- and three-way interactions between these as explanatory variables. We compared full models with reduced models to determine whether the inclusion of specific terms generated significantly better models (based on χ^2-test). We started by comparing a full model including trait, site, competition and all interactions with a model including all two-way interactions. If the model including the three-way interaction did not significantly improve the model, we proceeded with comparing the model including all two-way interactions with three models excluding each two-way interaction (Trait × Site, Trait × Competition, Site × Competition) to determine whether any of these interaction terms were significant. If none of the two-way interaction terms significantly improved the models, we proceeded by comparing a model excluding all interactions (only including Trait + Site + Competition) with three models excluding each variable (Trait, Site, Competition) to determine if any of these terms were significant. The

most parsimonious models (Table 1) were used to estimate selection across species and traits (Fig. 2-5) while results from full models are presented in Fig. S15.

Accounting for the potential effect of size on selection differentials

The overall size and age of an individual are likely to be correlated with the size of morphological traits (e.g. older and larger individuals are taller) and flowering phenology (e.g. larger plants are likely to flower earlier; Ollerton and Lack 1998; Valencia *et al.* 2016) but can also affect the probability of survival and potential for seed production. Many authors have argued that size is a more important predictor of plant life-history attributes such as mortality, flowering and seed production than age (Kirkpatrick 1984; Hanzawa and Kalisz 1993; Menges 2000) and that plant size often is correlated with age in perennial plants (Andrello *et al.* 2016; Troth *et al.* 2018, however see Perkins *et al.* 2006; Lauenroth and Adler 2008). Age effects were also partly accounted for here by excluding very large or small individuals when collecting individuals included in the experiment (2016).

Implementing block effects

For the model selection procedure performed to identify the most parsimonious models (see Material and methods), block (Fig. S1) was not implemented in the models as a random factor since the more complex models, such as the full model including the three-way interaction (Trait × Site × Competition), including block generated models with convergence issues. Block was however included when possible in final, most parsimonious, models used to estimate selection differentials, although implementing block generated singular fit for *T. badium* and *C. scheuchzeri*. For these cases, we first implemented a simplified version of the block effect, where

instead of 10 blocks (Fig. S1) block was divided into three sections representing the upper, middle and lower parts of the site (n = 3). Nonetheless, implementing block effects in this way still generated singular fits, and we therefore decided to exclude random effects for these cases.

Exploring year effects on traits, seed production and their interaction

To explore year effects on traits and seed production, we fitted linear models (lm for traits and seed production and glm for models including survival) for each species separately across warming/competitor treatments with traits/seed production/survival as response variables and year as explanatory variables. To explore year effects on the relationship between traits and seed production/survival, we fitted models with seed production/survival as response variables and year, trait and the two-way interactions between these as explanatory variables. We compared full models (Trait × Year) with reduced models (excluding the interaction, i.e. only year effect) to determine whether including year as a main or interactive effect generated significantly better models. P-values were based on F-tests for models including traits and seed production and χ^2-test for models including survival. For details on year effects, see Table S16.

Estimating global heritability for P. alpina

To explore the potential of traits to respond to selection in a case study among our focal species, we obtained estimates of heritability for one of the species, *P. alpina*, by relying on a relatedness matrix inferred from RAD-sequencing genotyping and implemented in restricted maximum-likelihood model (GREML) to quantify additive genetic variance. Because of the multifactorial nature of our experiment, heritability

was inferred after removing environmental effects simulated in the experiment and we term this “global heritability”. While this makes our estimates abstract in the sense that they do not relate to any specific site and warming/competition treatment, our goal was to assess whether trait expression could be underlain by additive genetic variance and make relative comparisons across traits and years.

Collection of leaf material

During June-July 2019, we collected and dried on silica gel leaves from 549 individuals of *P. alpina* from the field experiment as well as 60 additional individuals from the source population at 2200 m separated from each other by at least 2 m (total n = 609; for sample sizes from each treatment see Table S17). For these additional individuals, we measured stalk height, SLA and floral size in August 2019.

DNA extraction

Collected leaves were used to extract Genomic DNA was extracted with sbeadex™ mini plant kit (LGC Genomics Germany) using a 96 well KingFisher™ Flex Purification Systems (Thermo Fisher Scientific™) magnetic particle manipulator, following the manufacturers’ instructions. DNA concentration was quantified using Qubit fluorometer (Thermo Fisher Scientific™) and Spark 10M Multimode Microplate Reader (Tecan Trading AG, Switzerland) using the BR Assay kit (Thermo Fisher Scientific™).

Preparation of ddRAD libraries

Based on a modified version of the protocol by Peterson et al. (Peterson *et al.* 2012; see Westergaard et al. 2019) we prepared double digest restriction-site associated

DNA (ddRAD) libraries for 609 unique samples (including 15 duplicates of random individuals to control for correct indexing of individuals).

Digestion for an amount of 180 ng DNA per sample was performed where each sample was digested with 0.4 μl EcoRI-HF, 0.4 μl TaqI, 2.5 μl Smartcut Buffer (New England Biolabs, Inc., Ipswitch, MA, USA) and 2 μl water incubated under 37 C° followed by 65 C° under 2x 30 minutes period. To inactivate restriction enzymes of ECORI-HF incubation was followed by 20 minutes in 80 C°. The digest was then cooled to 4 C° before purified with AMPure beads (Beckman). The double digest was then ligated by adding 1.6 μl P2-biotin adapter, 3 μl T4 ligase buffer (10x), 1 μl T4 ligase NEB (400U/μl) and 3 μl water. 1 μl P1 Adapters were added to each sample and each sample was assigned a unique barcode (1- 48, i.e. 2x barcode per 96-well plate). The material was ligated 60 minutes at 23 C° followed by 65 C° for 10 minutes. The 48 uniquely barcoded samples were pooled and purified with AMPure beads (Beckman) before DNA concentrations were measured on a Qubit 2.0 device using the dsDBA BR Assay kit and size-selected (550 bp) with Dynabeads M-270 Streptavidin (Invitrogen). Unique combinations of 2 indexes were added to each 1-48 unique sample (i.e. 2 indexes per 96-well plate), representing a total of 7 sub-libraries. DNA was amplified using KAPA HiFi DNA Polymerase (PCR amplification, 7 cycles). Finally, the product was cleaned with AMPure beads (Beckman). DNA quantity of sub-libraries was estimated using Spark 10M Multimode Microplate Reader (Tecan Trading AG, Switzerland) using the BR Assay kit (Thermo Fisher Scientific™). Fragment size was determined using a 2200 Agilent Tapestation using High sensitivity D1000 ScreenTape. Sub-libraries were combined into 2 ddRAD

libraries containing 288 and 336 samples each. After filtering, we retained 10,987 SNPs for 554 unique individuals.

Libraries were sequenced in two lanes of 150-bp paired-end reads on an Illumina HiSeq 2500 at Novogene, UK.

dDocent workflow

The *de novo* assembly of the reference genome, mapping, variant calling and filtering was performed following the dDocent workflow (Puritz *et al.* 2014) to obtain SNPs used for estimates of relatedness. Firstly, the function "process_radtags" from Stacks v.2.41 (Catchen *et al.* 2013) was used to demultiplex samples.

For *de novo* assembling we used the RADseq assembly program Rainbow (Fu *et al.* 2012), where untrimmed, paired-end reads were reverse complemented and concatenated to forward reads as an initial step. Unique paired reads were identified and clusters of merged reads were assembled using a greedy algorithm (Pop and Salzberg 2008). The longest contig for each cluster was used as the representative reference sequence for that RAD locus and reference sequences were clustered based on overall sequence similarity (99 %), using the program CD-HIT v.4.6.8 (Chong *et al.* 2012). To optimize parameters, we maximized the remapping rate by varying the coverage of unique sequences within individuals.

To map reads to the reference contigs, we used BWA v.0.7.17 (Li and Durbin 2009). SNPs were called using FreeBayes v.1.3 (Garrison and Marth 2012). SNPs were then filtered using VCFtools v.0.1.16 and vcflib v.1.0.1 where we only kept SNPs that had

been successfully genotyped with a minimum quality score of 20, minimum mean depth of 3, mean depth of 10, minor allele count of 3, and minor allele frequency of 5%. We removed loci with more than 20% missing data per population. After filtering we obtained 10,987 SNPs for 554 unique individuals.

Estimating SNP-heritability

To estimate SNP-heritability (h^2_{SNP}), we computed the genomic relationship matrix (GRM) between individuals from the SNPs by using Genome-wide Complex Traits Analysis (GCTA; Yang *et al*. 2011). The GRM was then fitted in a genomic-relatedness-based restricted maximum-likelihood model (GREML) to estimate the amount of phenotypic variance in traits that was explained by additive genetic variation. Due to limited sample size, we did not have the statistical power to quantify heritability for each simulated warming level, competitor treatment or transplant treatment (individuals included/excluded in the experiment) separately. Instead, we quantified a 'global heritability' estimate for each trait across warming, competitor and transplant treatments (see Athanasiadis *et al*. 2020 for a similar approach). We fitted linear models in which phenotypic trait values were implemented as response variables and site (simulated warming), competition (present/absent) and competitor/soil origin (high/low elevation) as explanatory variables. An additional factor was included to account for whether individuals were sampled in the transplant experiment or the source/original population. When fitting models used to obtain residuals representing phenotypes in the GREML, models were fitted using Gaussian family distribution for all traits across all years, except for leaf dry weight for which we implemented models with Gamma family distribution. Note that for flowering time the term "transplantation" was excluded from the models as flowering

time only was recorded for individuals that were included in the transplant experiment while continuous phenology observations were not performed for individuals outside the transplant experiment.

By implementing the residuals from these models as phenotypes in the GREML, we accounted for the phenotypic variance explained by the experimental treatments and thus removed environmental effects simulated in the experiment. Heritability was estimated for the phenotypes measured for each year of the experiment (2017-2019) separately, to obtain year-specific values. Note that estimates of heritability obtained here are used to explore differences in the ability of traits to evolve across years. Estimates are therefore used for comparative purposes only and not to make predictions of evolutionary responses based on absolute heritability estimates.

For estimates of global heritability of traits for *P. alpina*, see Table S18.

Table S1. Information on species included in the experiment.

	A. alpestris	***T. badium***	***P. alpina***	***C. scheuchzeri***
**Family**	Fabaceae	Fabaceae	Plantaginaceae	Campanulaceae
**Pollinating agent**	Insect	Insect	Wind	Insect
**Growth form**	Branched stems	Branched stems	Vegetative basal rosettes	Single stem
**Flowers**	Aggregated in flower heads	Aggregated in flower heads	Single flowers aggregated on elongated stems	Stems with single flowers
**Distribution study area**	Limited to ≤2200 m	Occasionally >2200 m (c. 2000 m)	Occasionally >2200 m (c. 2000 m)	Limited to ≤2200 m

Table S2. Differences in absolute mean and variation of fitness and trait for *A. alpestris* across competitor and soil origins. Pairwise comparisons between competitor and soil origins (see Supplementary methods, "*Experimental design"*) of absolute means and variation of fitness and traits are based on Tukey HSD tests and analyses of heterogeneous variance, respectively. Note that differences in fitness/trait variation for *C. scheuchzeri* were assessed by performing Levene's test, as models fitted for analyses of heterogeneous variance failed to converge. False discovery rate (FDR) corrections of p-values for fitness/traits means (and variation for *C.* scheuchzeri) were performed across competitor/soil origin for each trait and species separately. Comparisons are based on most parsimonious models (Table 1) from which selection was estimated. Differences were considered significant at p < 0.05 and are indicated in bold for absolute means and variation. Note that differences in fitness/trait mean and variation are shown only for cases in which competition had an impact on selection.

A. alpestris	**Fitness mean**	**Fitness variation**	**Trait mean**	**Trait variation**
**STALK HEIGHT**				
Current vs. novel species	0.709	**0.001**	**< 0.001**	**< 0.001**
Alpine vs. low elevation soil	**0.013**	0.436	**< 0.001**	0.175
Current species vs. alpine soil	**< 0.001**	**< 0.001**	0.984	**0.003**
Novel species vs. low elevation soil	0.303	**< 0.001**	0.984	**0.032**
**SLA**				
Current vs. novel species	0.354	**0.001**	0.143	**0.004**
Alpine vs. low elevation soil	**< 0.001**	0.063	0.233	**< 0.001**
Current species vs. alpine soil	**< 0.001**	**< 0.001**	**< 0.001**	**< 0.001**
Novel species vs. low elevation soil	0.202	**< 0.001**	**< 0.001**	**< 0.001**
**FLOWERING TIME**				
Current vs. novel species	0.689	0.729	0.217	**0.04**
Alpine vs. low elevation soil	**0.019**	0.705	**< 0.001**	0.099
Current species vs. alpine soil	**< 0.001**	**< 0.001**	**0.031**	0.637
Novel species vs. low elevation soil	0.139	**< 0.001**	0.988	0.278

Table S3. Differences in absolute mean and variation of fitness and trait for *T. badium* across competitor and soil origins. See table text Table S2.

T. badium	**Fitness mean**	**Fitness variation**	**Trait mean**	**Trait variation**
**STALK HEIGHT**				
Current vs. novel species	**< 0.001**	0.157	0.741	0.099
Alpine vs. low elevation soil	**< 0.001**	**0.023**	**0.018**	0.941
Current species vs. alpine soil	0.169	**0.007**	**0.001**	0.397
Novel species vs. low elevation soil	0.994	0.264	**< 0.001**	**0.042**
**SLA**				
Current vs. novel species	**< 0.001**	**< 0.001**	0.723	0.489
Alpine vs. low elevation soil	**< 0.001**	**< 0.001**	0.476	**< 0.001**
Current species vs. alpine soil	0.911	**< 0.001**	**< 0.001**	0.135
Novel species vs. low elevation soil	**0.019**	**0.001**	**0.042**	**0.001**

Table S4. Differences in absolute mean and variation of fitness and trait for *P. alpina* across competitor and soil origins. See table text Table S2.

P. alpina	**Fitness mean**	**Fitness variation**	**Trait mean**	**Trait variation**
**STALK HEIGHT**				
Current vs. novel species	0.956	**0.006**	**0.001**	0.327
Alpine vs. low elevation soil	0.956	0.141	**0.042**	0.065
Current species vs. alpine soil	**< 0.001**	**0.002**	0.357	0.363
Novel species vs. low elevation soil	**< 0.001**	**< 0.001**	0.8	0.681
**SLA**				
Current vs. novel species	0.5	**< 0.001**	0.952	**0.027**
Alpine vs. low elevation soil	0.211	**0.008**	0.717	**0.015**
Current species vs. alpine soil	**< 0.001**	**< 0.001**	**< 0.001**	**< 0.001**
Novel species vs. low elevation soil	**< 0.001**	**< 0.001**	**< 0.001**	**< 0.001**

Table S5. Differences in absolute mean and variation of fitness and trait for *C. scheuchzeri* across competitor and soil origins. See table text Table S2.

C. scheuchzeri	**Fitness mean**	**Fitness variation**	**Trait mean**	**Trait variation**
**STALK HEIGHT**				
Current vs. novel species	0.574	**0.019**	0.999	0.305
Alpine vs. low elevation soil	0.403	0.349	0.709	0.234
Current species vs. alpine soil	**0.001**	**< 0.001**	0.709	0.1
Novel species vs. low elevation soil	**0.036**	**0.002**	0.709	**0.018**
**SLA**				
Current vs. novel species	0.389	**0.001**	0.569	0.49
Alpine vs. low elevation soil	**0.008**	0.8	0.95	0.189
Current species vs. alpine soil	**< 0.001**	**< 0.001**	**0.034**	**0.002**
Novel species vs. low elevation soil	**< 0.001**	**< 0.001**	**0.034**	0.858
**FLOWERING TIME**				
Current vs. novel species	0.894	0.17	0.997	0.719
Alpine vs. low elevation soil	0.867	0.267	0.079	**< 0.001**
Current species vs. alpine soil	**0.009**	**< 0.001**	0.997	0.974
Novel species vs. low elevation soil	0.311	0.058	0.997	**0.001**
**FLORAL SIZE**				
Current vs. novel species	0.81	**0.008**	1	0.409
Alpine vs. low elevation soil	0.81	0.542	1	0.71
Current species vs. alpine soil	0.235	**0.033**	1	0.114
Novel species vs. low elevation soil	0.418	0.08	1	0.427

Table S6. Pearson correlation coefficients of trait values between years (2017 vs. 2018 and 2018 vs. 2019). Correlations were considered significant when $p < 0.05$ and are indicated in bold.

	Comparisons	Pearson correlation coefficient
P. alpina		
Stalk height	2017 vs. 2018	**0.27**
Stalk height	2018 vs. 2019	**0.495**
SLA	2017 vs. 2018	**0.16**
SLA	2018 vs. 2019	**0.324**
Flowering time (Julian date)	2017 vs. 2018	**0.517**
Flowering time (Julian date)	2018 vs. 2019	**0.474**
A. alpestris		
Stalk height	2017 vs. 2018	0.005
Stalk height	2018 vs. 2019	0.041
SLA	2017 vs. 2018	**0.24**
SLA	2018 vs. 2019	**0.249**
Floral size	2017 vs. 2018	-0.004
Floral size	2018 vs. 2019	0.238
Flowering time (Julian day)	2017 vs. 2018	**0.744**
Flowering time (Julian day)	2018 vs. 2019	**0.855**
T. badium		
Stalk height	2017 vs. 2018	0.103
Stalk height	2018 vs. 2019	0.19
SLA	2017 vs. 2018	**0.264**
SLA	2018 vs. 2019	**0.252**
Flowering time (Julian day)	2017 vs. 2018	**0.675**
Flowering time (Julian day)	2018 vs. 2019	**0.493**
C. scheuchzeri		
Stalk height	2017 vs. 2018	**0.357**
Stalk height	2018 vs. 2019	**0.412**
SLA	2017 vs. 2018	**0.262**
SLA	2018 vs. 2019	**0.196**
Floral size	2017 vs. 2018	**0.435**
Floral size	2018 vs. 2019	0.231
Flowering time (Julian day)	2017 vs. 2018	-0.047
Flowering time (Julian day)	2018 vs. 2019	0.228

Table S7. Effects of size accounted for when estimating selection differentials for each trait and species. Estimates of size effects accounted for in linear models used to estimate selection differentials for each trait and species are shown as bootstrapped model coefficients. Size effects were considered significant when 95% of differences of bootstrapped selection differentials were not overlapping 0 and are indicated in bold. Note size effects only are shown for traits for which warming and competition significantly altered selection differentials (Table 1).

	Trait	Size effects
A. alpestris		
	Stalk height	**0.262** [0.221, 0.301]
	SLA	**0.454** [0.422, 0.486]
	Flowering time	**0.354** [0.314, 0.393]
T. badium		
	Stalk height	**0.273** [0.22, 0.325]
	SLA	**0.4** [0.359, 0.442]
	Flowering time	**0.268** [0.268, 0.319]
P. alpina		
	Stalk height	**0.143** [0.103, 0.182]
	SLA	**0.261** [0.233, 0.291]
C. scheuchzeri		
	Stalk height	**0.343** [0.262, 0.425]
	SLA	**0.423** [0.365, 0.482]
	Flowering time	**0.298** [0.194, 0.4]
	Floral size	**0.378** [0.289, 0.464]

Table S8. Pairwise differences in selection differentials between warming and competitor treatments and confidence intervals (CIs) for *A. alpestris*. Differences are based on pairwise comparisons between treatments of each selection differential based on bootstrapped model coefficients. Pairwise differences were considered significant when 95% of differences of bootstrapped selection differentials were not overlapping 0 and are indicated in bold.

A. alpestris	**Δ Selection differential**
**STALK HEIGHT**	
Warming – Competitors present	
4.85 vs. 3.05 °C	-0.071 [-0.171, 0.034]
4.85 vs. 1.65 °C	0.082 [-0.037, 0.202]
4.85 vs. 0 °C	0.129 [-0.033, 0.29]
3.05 vs. 1.65 °C	**0.152** [0.016, 0.285]
3.05 vs. 0 °C	**0.199** [0.022, 0.375]
1.65 vs. 0 °C	0.047 [-0.138, 0.229]
Warming – Competitors absent	
4.85 vs. 3.05 °C	-0.071 [-0.162, 0.025]
4.85 vs. 1.65 °C	0.083 [-0.031, 0.195]
4.85 vs. 0 °C	0.129 [-0.037, 0.294]
3.05 vs. 1.65 °C	**0.153** [0.036, 0.271]
3.05 vs. 0 °C	**0.2** [0.028, 0.367]
1.65 vs. 0 °C	0.047 [-0.134, 0.223]
Presence vs. absence of competitors	
4.85 °C	**-0.186** [-0.271, -0.102]
3.05 °C	**-0.186** [-0.299, -0.076]
1.65 °C	**-0.185** [-0.327, -0.044]
0 °C	-0.185 [-0.398, 0.028]
**SLA**	
Warming – Competitors present	
4.85 vs. 3.05 °C	**-0.154** [-0.255, -0.055]
4.85 vs. 1.65 °C	0.029 [-0.06, 0.118]
4.85 vs. 0 °C	0.05 [-0.161, 0.256]
3.05 vs. 1.65 °C	**0.183** [0.074, 0.295]
3.05 vs. 0 °C	0.203 [-0.018, 0.42]
1.65 vs. 0 °C	0.02 [-0.195, 0.235]

Warming – Competitors absent	
4.85 vs. 3.05 °C	0.09 [-0.027, 0.211]
4.85 vs. 1.65 °C	-0.068 [-0.226, 0.093]
4.85 vs. 0 °C	0.092 [-0.142, 0.322]
3.05 vs. 1.65 °C	-0.158 [-0.337, 0.025]
3.05 vs. 0 °C	0.002 [-0.251, 0.248]
1.65 vs. 0 °C	0.16 [-0.121, 0.44]
Presence vs. absence of competitors	
4.85 °C	0.026 [-0.053, 0.103]
3.05 °C	**0.27 [0.141, 0.402]**
1.65 °C	-0.071 [-0.232, 0.096]
0 °C	0.069 [-0.228, 0.364]
**FLOWERING TIME**	
Warming – Competitors present	
4.85 vs. 3.05 °C	-0.144 [-0.367, 0.082]
4.85 vs. 1.65 °C	-0.243 [-0.516, 0.03]
4.85 vs. 0 °C	**-0.302** [-0.512, -0.097]
3.05 vs. 1.65 °C	-0.099 [-0.388, 0.193]
3.05 vs. 0 °C	-0.158 [-0.394, 0.077]
1.65 vs. 0 °C	-0.06 [-0.343, 0.23]
Warming – Competitors absent	
4.85 vs. 3.05 °C	-0.144 [-0.352, 0.072]
4.85 vs. 1.65 °C	-0.243 [-0.513, 0.027]
4.85 vs. 0 °C	**-0.303** [-0.497, -0.095]
3.05 vs. 1.65 °C	-0.099 [-0.395, 0.202]
3.05 vs. 0 °C	-0.159 [-0.395, 0.081]
1.65 vs. 0 °C	-0.06 [-0.336, 0.227]
Presence vs. absence of competitors	
4.85 °C	0.036 [-0.152, 0.22]
3.05 °C	0.036 [-0.205, 0.279]
1.65 °C	0.036 [-0.3, 0.361]
0 °C	0.035 [-0.185, 0.255]

Table S9. Pairwise differences in selection differentials between warming and competitor treatments and confidence intervals (CIs) for *T. badium*. See table text Table S8.

T. badium	**Δ Selection differential**
**STALK HEIGHT**	
Warming – Competitors present	
4.85 vs. 3.05 °C	0.052 [-0.127, 0.242]
4.85 vs. 1.65 °C	-0.062 [-0.251, 0.127]
4.85 vs. 0 °C	0.161 [-0.17, 0.511]
3.05 vs. 1.65 °C	-0.114 [-0.316, 0.092]
3.05 vs. 0 °C	0.109 [-0.241, 0.458]
1.65 vs. 0 °C	0.223 [-0.123, 0.56]
Warming – Competitors absent	
4.85 vs. 3.05 °C	-0.195 [-0.424, 0.028]
4.85 vs. 1.65 °C	**-0.734** [-0.979, -0.495]
4.85 vs. 0 °C	**-0.369** [-0.652, -0.093]
3.05 vs. 1.65 °C	**-0.538** [-0.754, -0.324]
3.05 vs. 0 °C	-0.174 [-0.433, 0.079]
1.65 vs. 0 °C	**0.364** [0.102, 0.629]
Presence vs. absence of competitors	
4.85 °C	**0.293** [0.077, 0.509]
3.05 °C	0.045 [-0.155, 0.238]
1.65 °C	**-0.379** [-0.594, -0.161]
0 °C	-0.238 [-0.625, 0.139]
**SLA**	
Warming	
4.85 vs. 3.05 °C	**-0.15** [-0.268, -0.03]
4.85 vs. 1.65 °C	**-0.125** [-0.232, -0.013]
4.85 vs. 0 °C	0.091 [-0.055, 0.23]
3.05 vs. 1.65 °C	0.026 [-0.074, 0.125]
3.05 vs. 0 °C	**0.241** [0.104, 0.379]
1.65 vs. 0 °C	**0.215** [0.089, 0.341]
**FLOWERING TIME**	
Warming	
4.85 vs. 3.05 °C	0.251 [-0.02, 0.525]
4.85 vs. 1.65 °C	-0.017 [-0.262, 0.228]
4.85 vs. 0 °C	**-0.338** [-0.525, -0.15]
3.05 vs. 1.65 °C	-0.268 [-0.571, 0.037]
3.05 vs. 0 °C	**-0.588** [-0.848, -0.325]
1.65 vs. 0 °C	**-0.321** [-0.554, -0.096]

Table S10. Pairwise differences in selection differentials between warming and competitor treatments and confidence intervals (CIs) for *P. alpina*. See table text Table S8.

P. alpina	**Δ Selection differential**
**STALK HEIGHT**	
Warming – Competitors present	
4.85 vs. 3.05 °C	0.036 [-0.093, 0.167]
4.85 vs. 1.65 °C	**-0.194** [-0.34, -0.05]
4.85 vs. 0 °C	0.014 [-0.291, 0.311]
3.05 vs. 1.65 °C	**-0.229** [-0.356, -0.101]
3.05 vs. 0 °C	-0.022 [-0.315, 0.273]
1.65 vs. 0 °C	0.208 [-0.086, 0.501]
Warming – Competitors absent	
4.85 vs. 3.05 °C	**-0.111** [-0.215, -0.005]
4.85 vs. 1.65 °C	-0.085 [-0.199, 0.031]
4.85 vs. 0 °C	0.043 [-0.133, 0.219]
3.05 vs. 1.65 °C	0.026 [-0.092, 0.148]
3.05 vs. 0 °C	**-0.111** [-0.215, -0.005]
1.65 vs. 0 °C	0.128 [-0.056, 0.314]
Presence vs. absence of competitors	
4.85 °C	**-0.191** [-0.317, -0.064]
3.05 °C	**-0.338** [-0.445, -0.23]
1.65 °C	-0.082 [-0.217, 0.058]
0 °C	-0.162 [-0.482, 0.18]
**SLA**	
Warming	
4.85 vs. 3.05 °C	**-0.063** [-0.122, -0.006]
4.85 vs. 1.65 °C	0.026 [-0.053, 0.103]
4.85 vs. 0 °C	**-0.117** [-0.218, -0.016]
3.05 vs. 1.65 °C	**0.089** [0.011, 0.166]
3.05 vs. 0 °C	-0.054 [-0.154, 0.049]
1.65 vs. 0 °C	**-0.143** [-0.253, -0.03]

Table S11. Pairwise differences in selection differentials between warming and competitor treatments and confidence intervals (CIs) for *C. scheuchzeri*. See table text Table S8.

C. scheuchzeri	**Δ Selection differential**
**STALK HEIGHT**	
Warming – Competitors present	
4.85 vs. 3.05 °C	0.172 [-0.13, 0.464]
4.85 vs. 1.65 °C	0.108 [-0.474, 0.687]
4.85 vs. 0 °C	0.102 [-0.484, 0.7]
3.05 vs. 1.65 °C	-0.064 [-0.669, 0.548]
3.05 vs. 0 °C	-0.07 [-0.683, 0.555]
1.65 vs. 0 °C	-0.006 [-0.798, 0.79]
Warming – Competitors absent	
4.85 vs. 3.05 °C	0.066 [-0.178, 0.321]
4.85 vs. 1.65 °C	**0.854** [0.558, 1.159]
4.85 vs. 0 °C	**0.583** [0.229, 0.928]
3.05 vs. 1.65 °C	**0.788** [0.509, 1.076]
3.05 vs. 0 °C	**0.517** [0.185, 0.852]
1.65 vs. 0 °C	-0.271 [-0.647, 0.097]
Presence vs. absence of competitors	
4.85 °C	**-0.448** [-0.703, -0.199]
3.05 °C	**-0.554** [-0.854, -0.256]
1.65 °C	0.033 [-0.618, 0.659]
0 °C	0.299 [-0.312, 0.887]
**SLA**	
Warming – Competitors present	
4.85 vs. 3.05 °C	-0.105 [-0.308, 0.103]
4.85 vs. 1.65 °C	-0.064 [-0.24, 0.111]
4.85 vs. 0 °C	-0.089 [-0.286, 0.106]
3.05 vs. 1.65 °C	0.04 [-0.148, 0.231]
3.05 vs. 0 °C	0.015 [-0.201, 0.221]
1.65 vs. 0 °C	-0.025 [-0.212, 0.159]
Warming – Competitors absent	
4.85 vs. 3.05 °C	0.134 [-0.067, 0.338]
4.85 vs. 1.65 °C	**-0.285** [-0.519, -0.051]
4.85 vs. 0 °C	**-0.357** [-0.645, -0.07]
3.05 vs. 1.65 °C	**-0.419** [-0.651, -0.19]
3.05 vs. 0 °C	**-0.491** [-0.786, -0.194]
1.65 vs. 0 °C	-0.071 [-0.376, 0.234]

Presence vs. absence of competitors	
4.85 °C	**0.222** [0.025, 0.415]
3.05 °C	**0.46** [0.251, 0.664]
1.65 °C	0.001 [-0.2, 0.213]
0 °C	-0.046 [-0.341, 0.246]
**FLOWERING TIME**	
Warming – Competitors present	
4.85 vs. 3.05 °C	0.646 [-0.211, 1.515]
4.85 vs. 1.65 °C	-0.043 [-0.543, 0.469]
4.85 vs. 0 °C	-0.094 [-1.002, 0.814]
3.05 vs. 1.65 °C	-0.689 [-1.588, 0.224]
3.05 vs. 0 °C	-0.74 [-1.883, 0.439]
1.65 vs. 0 °C	-0.05 [-0.976, 0.884]
Warming – Competitors absent	
4.85 vs. 3.05 °C	0.065 [-0.679, 0.766]
4.85 vs. 1.65 °C	**0.869** [0.01, 1.685]
4.85 vs. 0 °C	-0.355 [-1.029, 0.283]
3.05 vs. 1.65 °C	**0.804** [0.133, 1.451]
3.05 vs. 0 °C	**-0.42** [-0.82, -0.02]
1.65 vs. 0 °C	**-1.224** [-1.805, -0.627]
Presence vs. absence of competitors	
4.85 °C	0.612 [-0.046, 1.31]
3.05 °C	0.031 [-0.847, 0.931]
1.65 °C	**1.525** [0.821, 2.224]
0 °C	0.351 [-0.549, 1.22]
**FLORAL SIZE**	
Presence vs. absence of competitors	**-0.532** [-0.761, -0.295]

Table S12. Differences in absolute mean and variation of fitness and trait for *A. alpestris*. Pairwise comparisons between warming levels (per competition treatment) and competitor treatments (per warming level) of absolute means and variation of fitness and traits are based on Tukey HSD tests and analyses of heterogeneous variance, respectively. Differences were considered significant at p < 0.05 and are indicated in bold. Cases where significant differences in fitness/trait mean/variation could be associated with significant shifts in selection are highlighted in grey (see Results).

A. alpestris	**Fitness mean**	**Fitness variation**	**Trait mean**	**Trait variation**
**STALK HEIGHT**				
Warming – Competitors present				
4.85 vs. 3.05 °C	1	0.337	**0.057**	**0.02**
4.85 vs. 1.65 °C	1	0.484	0.085	**0.001**
4.85 vs. 0 °C	0.598	**0.007**	1	0.058
3.05 vs. 1.65 °C	1	0.86	1	0.185
3.05 vs. 0 °C	0.73	**0.043**	1	0.781
1.65 vs. 0 °C	1	**0.035**	1	0.427
Warming – Competitors absent				
4.85 vs. 3.05 °C	0.73	0.576	0.814	0.465
4.85 vs. 1.65 °C	**0.001**	0.249	1	0.395
4.85 vs. 0 °C	**0.004**	**0.006**	1	**< 0.001**
3.05 vs. 1.65 °C	0.298	0.125	1	0.162
3.05 vs. 0 °C	0.439	**0.003**	1	**< 0.001**
1.65 vs. 0 °C	1	0.103	1	**< 0.001**
Presence vs. absence of competitors				
4.85 °C	**< 0.001**	**< 0.001**	**0.004**	**< 0.001**
3.05 °C	**< 0.001**	**< 0.001**	1	0.873
1.65 °C	**0.015**	**< 0.001**	1	**0.008**
0 °C	**0.003**	**< 0.001**	1	**0.01**

**SLA**				
Warming – Competitors present				
4.85 vs. 3.05 °C	**0.022**	0.653	**0.002**	**< 0.001**
4.85 vs. 1.65 °C	**< 0.001**	**0.038**	**< 0.001**	**0.011**
4.85 vs. 0 °C	**< 0.001**	**< 0.001**	**< 0.001**	**< 0.001**
3.05 vs. 1.65 °C	0.963	**0.013**	1	0.666
3.05 vs. 0 °C	0.677	**< 0.001**	1	**< 0.001**
1.65 vs. 0 °C	1	**< 0.001**	1	**< 0.001**
Warming – Competitors absent				
4.85 vs. 3.05 °C	**< 0.001**	0.38	0.999	**< 0.001**
4.85 vs. 1.65 °C	**< 0.001**	0.991	0.179	**< 0.001**
4.85 vs. 0 °C	**< 0.001**	**0.033**	0.999	**< 0.001**
3.05 vs. 1.65 °C	**< 0.001**	0.39	**0.009**	**< 0.001**
3.05 vs. 0 °C	0.802	**0.005**	0.415	**< 0.001**
1.65 vs. 0 °C	0.364	**0.037**	0.999	0.251
Presence vs. absence of competitors				
4.85 °C	**< 0.001**	**< 0.001**	**< 0.001**	0.538
3.05 °C	**< 0.001**	**< 0.001**	0.397	**0.003**
1.65 °C	**< 0.001**	**< 0.001**	**< 0.001**	**< 0.001**
0 °C	**< 0.001**	**< 0.001**	0.185	0.317
**FLOWERING TIME**				
Warming – Competitors present				
4.85 vs. 3.05 °C	0.411	0.801	**< 0.001**	0.25
4.85 vs. 1.65 °C	0.607	0.422	**< 0.001**	0.457
4.85 vs. 0 °C	**0.031**	**0.041**	**< 0.001**	**0.001**
3.05 vs. 1.65 °C	1	0.337	**< 0.001**	0.1
3.05 vs. 0 °C	0.836	**0.033**	**< 0.001**	**0.038**
1.65 vs. 0 °C	0.836	0.159	**0.023**	**0.001**
Warming – Competitors absent				
4.85 vs. 3.05 °C	0.836	0.809	**< 0.001**	**0.02**
4.85 vs. 1.65 °C	**< 0.001**	0.757	**< 0.001**	**0.002**
4.85 vs. 0 °C	**< 0.001**	**0.049**	**< 0.001**	0.175
3.05 vs. 1.65 °C	**0.002**	0.623	**< 0.001**	0.38
3.05 vs. 0 °C	**0.008**	0.097	**< 0.001**	**0.001**
1.65 vs. 0 °C	1	**0.041**	**< 0.001**	**< 0.001**
Presence vs. absence of competitors				
4.85 °C	**< 0.001**	**0**	0.116	0.273
3.05 °C	**< 0.001**	**0**	1	0.026
1.65 °C	**0.026**	**0**	1	0.301
0 °C	**0.001**	**0.001**	1	0.2

Table S13. Differences in absolute mean and variation of fitness and trait for *T. badium*. See table text Table S12.

T. badium	Fitness mean	Fitness variation	Trait mean	Trait variation
**STALK HEIGHT**				
Warming – Competitors present				
4.85 vs. 3.05 °C	**< 0.001**	0.312	0.191	0.522
4.85 vs. 1.65 °C	**0.002**	0.079	0.749	0.078
4.85 vs. 0 °C	1	0.353	1	**0.019**
3.05 vs. 1.65 °C	1	0.482	1	0.284
3.05 vs. 0 °C	**0.025**	0.101	**0.031**	0.056
1.65 vs. 0 °C	0.262	**0.033**	0.157	0.219
Warming – Competitors absent				
4.85 vs. 3.05 °C	0.284	0.065	**0.029**	0.148
4.85 vs. 1.65 °C	0.049	**< 0.001**	**< 0.001**	0.507
4.85 vs. 0 °C	1	0.312	**0.013**	0.463
3.05 vs. 1.65 °C	1	**0.012**	1	0.435
3.05 vs. 0 °C	1	0.422	1	**0.031**
1.65 vs. 0 °C	1	**0.002**	1	0.167
Presence vs. absence of competitors				
4.85 °C	**0.003**	0.177	**< 0.001**	**0.012**
3.05 °C	1	**0.031**	**0.001**	0.641
1.65 °C	**0.037**	**< 0.001**	0.713	0.835
0 °C	**0.01**	**0.011**	1	0.992
**SLA**				
Warming				
4.85 vs. 3.05 °C	**< 0.001**	**< 0.001**	0.399	0.097
4.85 vs. 1.65 °C	**< 0.001**	**< 0.001**	**< 0.001**	0.689
4.85 vs. 0 °C	0.889	**< 0.001**	0.125	0.111
3.05 vs. 1.65 °C	0.286	**0.034**	**0.001**	**0.017**
3.05 vs. 0 °C	**0.002**	0.934	0.733	0.885
1.65 vs. 0 °C	**< 0.001**	0.085	0.125	**0.031**
**FLOWERING TIME**				
Warming				
4.85 vs. 3.05 °C	**< 0.001**	0.19	**< 0.001**	**< 0.001**
4.85 vs. 1.65 °C	**0.001**	**0.002**	**< 0.001**	**< 0.001**
4.85 vs. 0 °C	0.259	0.474	**< 0.001**	**0.013**
3.05 vs. 1.65 °C	0.718	0.064	**< 0.001**	0.364
3.05 vs. 0 °C	0.112	0.623	**< 0.001**	**< 0.001**
1.65 vs. 0 °C	0.453	**0.032**	**< 0.001**	**< 0.001**

Table S14. Differences in absolute mean and variation of fitness and trait for *P. alpina*. See table text Table S12.

P. alpina	Fitness mean	Fitness variation	Trait mean	Trait variation
**STALK HEIGHT**				
Warming – Competitors present				
4.85 vs. 3.05 °C	0.983	0.141	0.367	0.188
4.85 vs. 1.65 °C	**0.009**	**< 0.001**	1	0.131
4.85 vs. 0 °C	0.983	0.311	1	0.573
3.05 vs. 1.65 °C	0.119	**< 0.001**	1	**0.002**
3.05 vs. 0 °C	0.887	0.091	1	0.225
1.65 vs. 0 °C	0.072	**0.002**	1	0.858
Warming – Competitors absent				
4.85 vs. 3.05 °C	**< 0.001**	0.092	1	0.094
4.85 vs. 1.65 °C	**< 0.001**	**0.028**	**0.017**	**0.006**
4.85 vs. 0 °C	0.462	**0.009**	0.373	**< 0.001**
3.05 vs. 1.65 °C	0.919	0.585	**< 0.001**	0.253
3.05 vs. 0 °C	0.088	**< 0.001**	**0.027**	**0.002**
1.65 vs. 0 °C	0.745	**< 0.001**	1	**0.032**
Presence vs. absence of competitors				
4.85 °C	**< 0.001**	**< 0.001**	1	0.788
3.05 °C	**< 0.001**	**< 0.001**	**< 0.001**	**0.005**
1.65 °C	**< 0.001**	**< 0.001**	1	0.687
0 °C	**< 0.001**	**0.007**	1	0.143
**SLA**				
Warming				
4.85 vs. 3.05 °C	**< 0.001**	**< 0.001**	0.399	**0.035**
4.85 vs. 1.65 °C	**< 0.001**	**0.023**	**< 0.001**	**< 0.001**
4.85 vs. 0 °C	0.889	0.051	0.125	**< 0.001**
3.05 vs. 1.65 °C	0.286	**0.035**	**0.001**	**< 0.001**
3.05 vs. 0 °C	**0.002**	0.097	0.733	**< 0.001**
1.65 vs. 0 °C	**< 0.001**	0.963	0.125	0.456

Table S15. Differences in absolute mean and variation of fitness and trait for *C. scheuchzeri*. See table text Table S12.

C. scheuchzeri	Fitness mean	Fitness variation	Trait mean	Trait variation
**STALK HEIGHT**				
Warming – Competitors present				
4.85 vs. 3.05 °C	1	0.41	1	0.701
4.85 vs. 1.65 °C	1	**< 0.001**	1	**< 0.001**
4.85 vs. 0 °C	1	**< 0.001**	1	0.052
3.05 vs. 1.65 °C	1	**< 0.001**	1	**< 0.001**
3.05 vs. 0 °C	1	**< 0.001**	1	0.113
1.65 vs. 0 °C	1	**< 0.001**	1	0.212
Warming – Competitors absent				
4.85 vs. 3.05 °C	1	0.265	1	0.73
4.85 vs. 1.65 °C	1	0.27	1	0.335
4.85 vs. 0 °C	1	0.694	1	**0.002**
3.05 vs. 1.65 °C	**0.04**	0.96	1	0.176
3.05 vs. 0 °C	0.721	0.442	1	**< 0.001**
1.65 vs. 0 °C	1	0.438	1	**0.038**
Presence vs. absence of competitors				
4.85 °C	**< 0.001**	**< 0.001**	1	**0.025**
3.05 °C	0.728	**0.008**	1	0.239
1.65 °C	**< 0.001**	**< 0.001**	1	**0.032**
0 °C	**0.003**	**< 0.001**	1	0.398
**SLA**				
Warming – Competitors present				
4.85 vs. 3.05 °C	1	0.435	1	0.252
4.85 vs. 1.65 °C	1	**< 0.001**	1	0.734
4.85 vs. 0 °C	1	**< 0.001**	1	0.333
3.05 vs. 1.65 °C	1	**< 0.001**	1	0.36
3.05 vs. 0 °C	1	**< 0.001**	1	0.841
1.65 vs. 0 °C	1	0.644	1	0.473
Warming – Competitors absent				
4.85 vs. 3.05 °C	0.18	0.074	1	0.092
4.85 vs. 1.65 °C	1	0.729	1	**0.035**
4.85 vs. 0 °C	1	0.338	1	**< 0.001**
3.05 vs. 1.65 °C	0.143	0.119	1	0.577
3.05 vs. 0 °C	0.322	0.391	1	**0.003**
1.65 vs. 0 °C	1	0.504	1	**0.017**
Presence vs. absence of competitors				
4.85 °C	**< 0.001**	**< 0.001**	1	0.898
3.05 °C	**< 0.001**	**< 0.001**	1	0.502
1.65 °C	**< 0.001**	**< 0.001**	1	**0.029**
0 °C	**< 0.001**	**< 0.001**	1	**< 0.001**

**FLOWERING TIME**				
Warming – Competitors present				
4.85 vs. 3.05 °C	1	0.488	1	0.074
4.85 vs. 1.65 °C	1	**< 0.001**	1	0.452
4.85 vs. 0 °C	1	**< 0.001**	**< 0.001**	0.746
3.05 vs. 1.65 °C	1	**< 0.001**	1	0.24
3.05 vs. 0 °C	1	**< 0.001**	**< 0.001**	0.343
1.65 vs. 0 °C	1	0.948	**< 0.001**	0.923
Warming – Competitors absent				
4.85 vs. 3.05 °C	0.506	0.17	1	**0.035**
4.85 vs. 1.65 °C	1	0.322	1	0.993
4.85 vs. 0 °C	1	0.878	**< 0.001**	**< 0.001**
3.05 vs. 1.65 °C	0.567	0.718	1	**0.024**
3.05 vs. 0 °C	1	0.095	**< 0.001**	**0.001**
1.65 vs. 0 °C	1	0.215	**< 0.001**	**< 0.001**
Presence vs. absence of competitors				
4.85 °C	**0.001**	**< 0.001**	1	**< 0.001**
3.05 °C	0.567	0.072	1	0.85
1.65 °C	**< 0.001**	**< 0.001**	1	**< 0.001**
0 °C	0.194	**< 0.001**	0.119	0.995
**FLORAL SIZE**				
Presence vs. absence of competitors	**< 0.001**	**< 0.001**	0.137	**0.001**

Table S16. Year effects on fitness/traits and relationship between seed production/survival and traits. P-values from F-tests for year effects on fitness/traits and the relationship between seed production and traits are shown in parenthesis. χ^2 tests were used to test year effects on survival and the relationship between traits and survival. If interactions were significant, p-values for main effects were not calculated. Effects were considered significant at p < 0.05.

Species	Trait name	Effect trait/fitness	Effect relationship seed production and trait	Effect relationship survival and trait
A. alpestris	Seed production	**Year** (0.001)	NA	NA
	Survival	**-**	NA	NA
	Flowering probability	**Year** (< 0.001)	NA	NA
	Stalk height	**Year** (<0.001)	**Trait x Year** (< 0.001)	**-**
	SLA	**Year** (0.003)	**Year** (<0.001)	**Trait + Year** (< 0.001)
	Flowering time	**Year** (< 0.001)	**Year** (< 0.001)	**-**
	Floral size	**-**	**Year** (< 0.001)	**-**
T. badium	Seed production	**-**	NA	NA
	Survival	**-**	NA	NA
	Flowering probability	**Year** (< 0.001)	NA	NA
	Stalk height	**Year** (< 0.001)	**-**	**Trait x Year** (0.041)
	SLA	**Year** (< 0.001)	**-**	**-**
	Flowering time	**Year** (< 0.001)	**-**	**-**
P. alpina	Seed production	**-**	NA	NA
	Survival	**Year** (< 0.001)	NA	NA
	Flowering probability	**-**	NA	NA

Stalk height	**Year** (< 0.001)	**Trait x Year** (< 0.001)	**Year** (< 0.001)	Stalk height
SLA	**Year** (< 0.001)	**-**	**Year** (< 0.001)	SLA
Flowering time	**Year** (< 0.001)	**-**	**Trait x Year** (< 0.001)	Flowering time
C. scheuchzeri	Seed production	**Year** (0.02)	NA	NA
	Survival	**-**	NA	NA
	Flowering probability	**Year** (< 0.001)	NA	NA
	Stalk height	**Year** (< 0.001)	-	**Trait x Year** (< 0.001)
	SLA	**Year** (< 0.001)	-	**Trait + Year** (< 0.001)
	Flowering time	**Year** (< 0.001)	**Trait + Year** (0.001)	**Trait x Year** (< 0.001)
	Floral size	**Year** (0.012)	**Trait + Year** (0.003)	**Trait + Year** (0.024)

Table S17. Sample size of individuals used for ddRAD preparation and heritability estimation for *P. alpina*. Sample sizes from each site and competitor/soil origin of the total number of individuals (554 after filtering and quality check, see Supplementary Methods for details) used to estimate heritability.

	1400 m	1750 m	1950 m	2200 m
**High elevation community**	35	31	39	34
**Low elevation community**	15	39	44	-
**High elevation soil**	39	36	37	38
**Low elevation soil**	31	39	40	-
**Individuals outside experiment**	-	-	-	57

Table S18. Global (SNP) heritability estimates (+/- 95% confidence intervals) for traits of *P. alpina*. Effects were considered significant when 95% CIs do not overlap 0 and are indicated in bold.

Trait	2017	2018	2019
Stalk height	< 0.01 [-1.324, 1.324]	0.316 [-0.068, 0.7]	0.087 [-0.282, 0.456]
Specific Leaf Area	0.029 [-0.276, 0.334]	< 0.01 [-0.25, 0.25]	**0.336 [0.078, 0.595]**
Flowering time	< 0.01 [-1.885, 1.885]	0.239 [-0.154, 0.631]	0.22 [-0.132, 0.573]

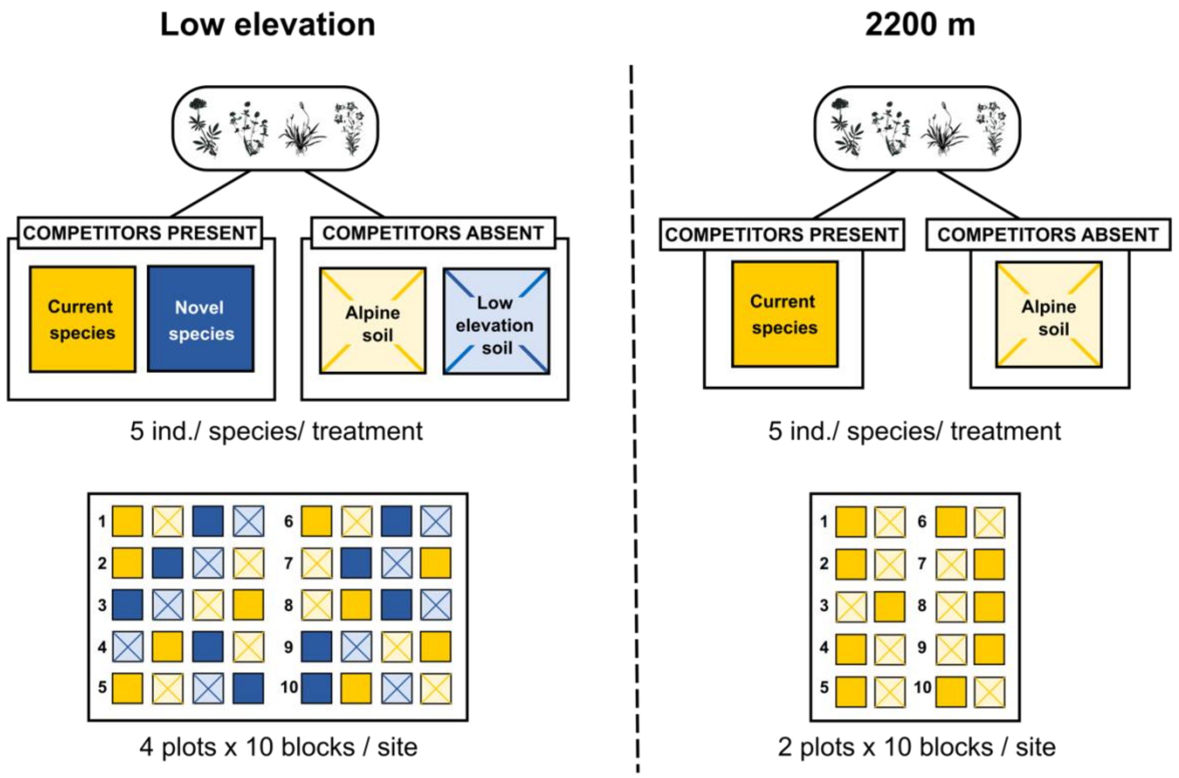

Figure S1. Details experimental set-up and block design. At each lower elevation site (1400, 1750, 1950 m), focal species were planted into plots where they interacted with competitors ("Competitors present"), consisting of plots with current and novel species communities. Focal species were also planted into plots where competitors were absent ("Competitors absent"; high or low elevation soils). Each treatment was replicated 10 times in blocks (marked 1-10 in figure), with one plot of each treatment per block. At the 2200 m site, focal species were only planted into plots representing current species ("Competitors present") and high elevation soil ("Competitors absent").

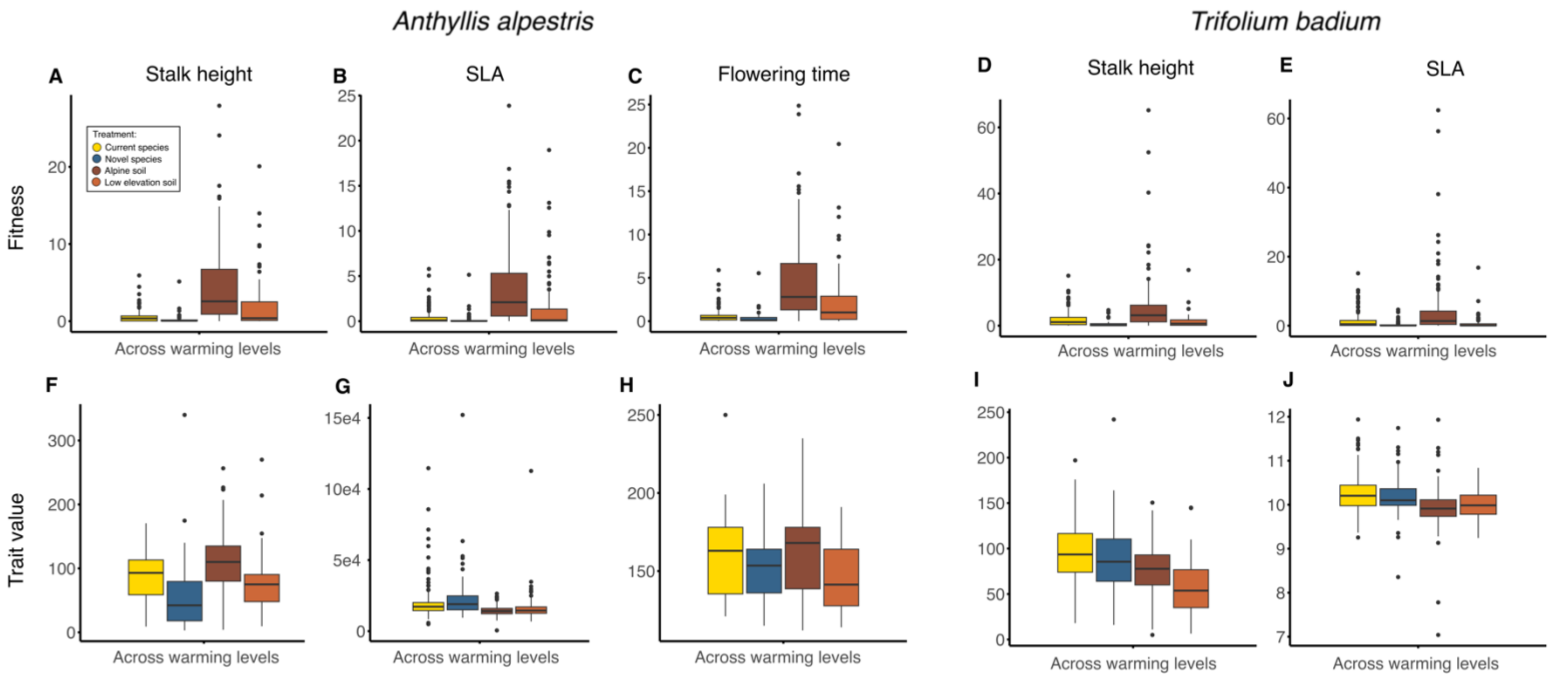

Figure S2. Distribution of fitness (A-E) and phenotypic traits (F-J) across competitor/soil origins for *A. alpestris* and *T. badium.* Trait/fitness are shown on the x-axis for current species (yellow), novel species (blue), alpine soil (dark brown) and low elevation soil (orange). Note that corresponding trait/fitness distributions across competitor/soil origins are not estimated for flowering time for *T. badium* as only warming had a significant effect on selection for this case (Table 1). For differences in absolute means and variation between competitor and soil origins see Table S2-3.

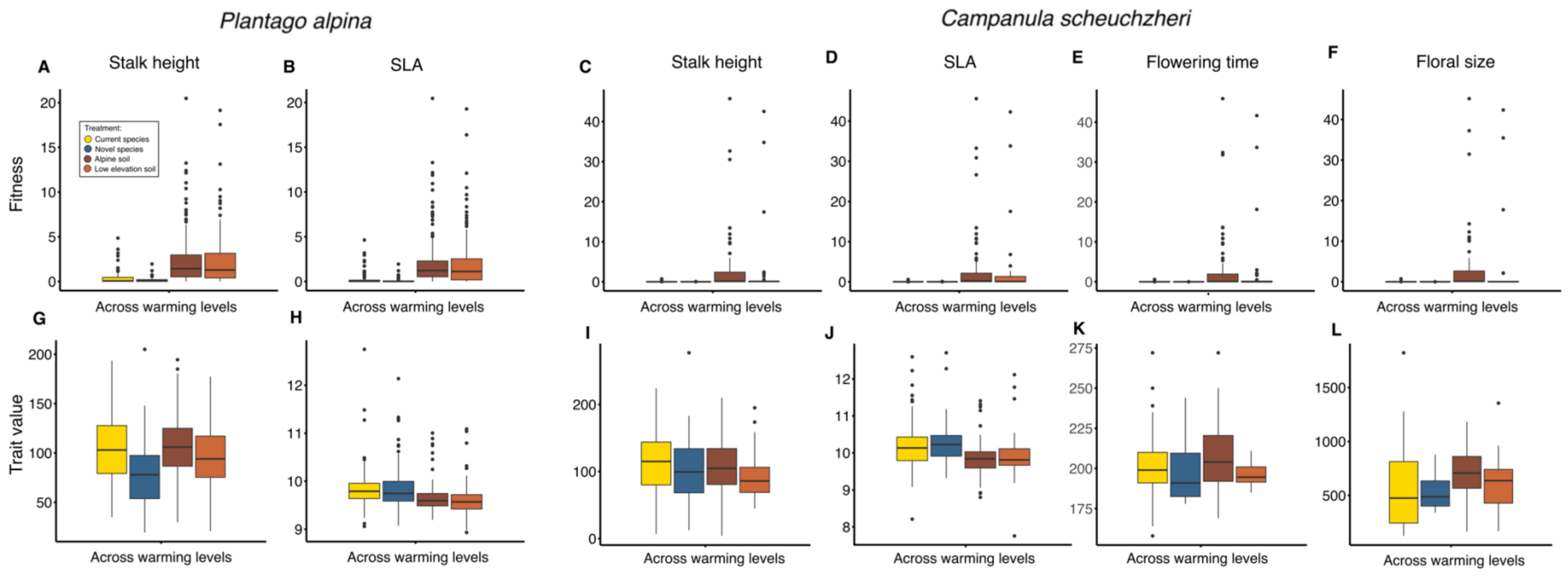

Figure S3. Distribution of fitness (A-F) and phenotypic traits (G-L) across competitor/soil origins for *P. alpina* and *C. scheuchzeri*. Trait/fitness values are shown on the x-axis for current species (yellow), novel species (blue), alpine soil (dark brown) and low elevation soil (orange). For differences in absolute means and variation between competitor and soil origins see Table S4-5.

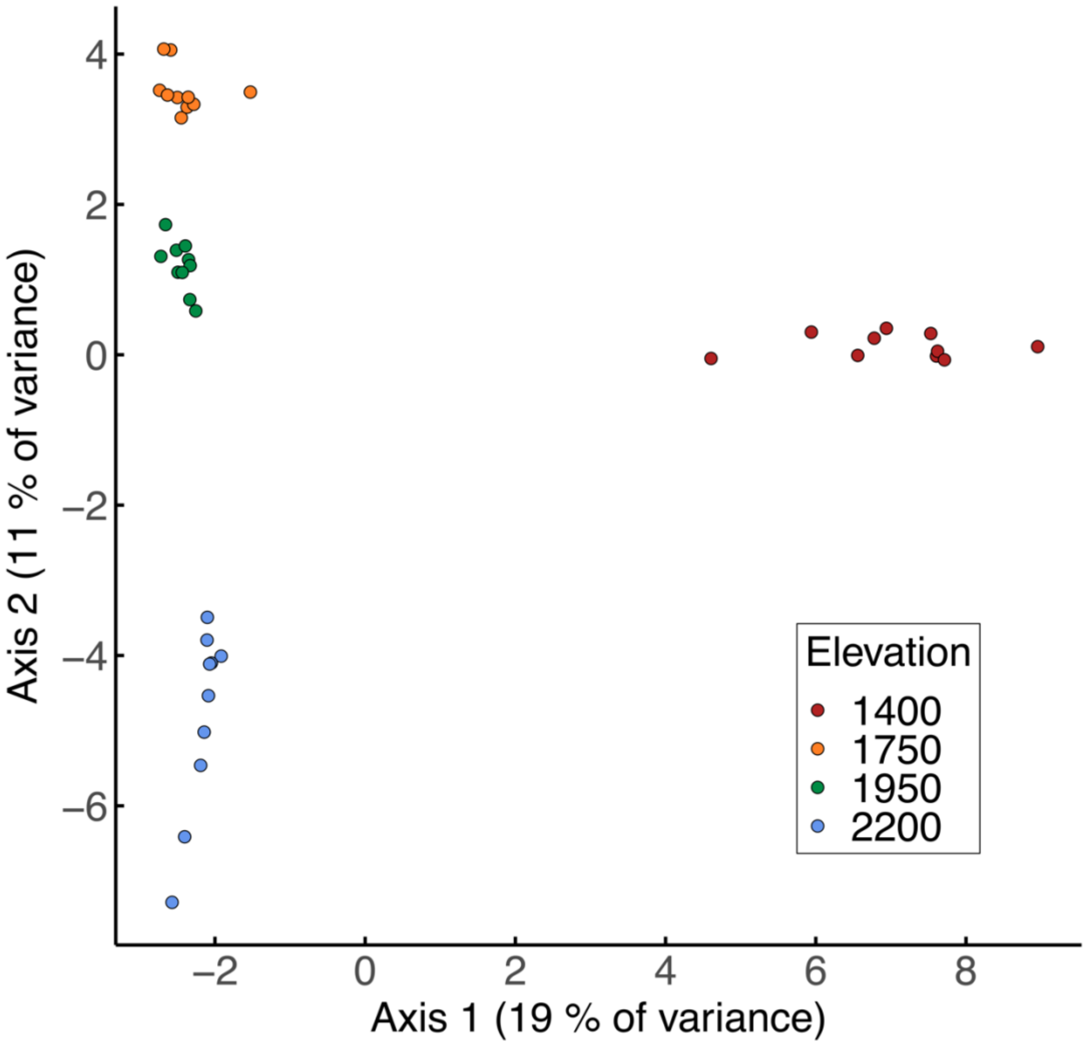

Figure S4. Plant community composition at each field site (four elevations between 1400 and 2200 m). Data show results of a principle components analysis (PCA) of floristic composition based on relative cover of vascular plant species within the 10 replicate "home site" turfs at each field site in the first year of the study (2017). The species abundances were used to calculate the relative cover of species within home-site turfs at each elevation. The relative cover of all species across sites was implemented into a PCA where the values were scaled. PCA scores of each turf were obtained and the figure shows the first and second principal components.

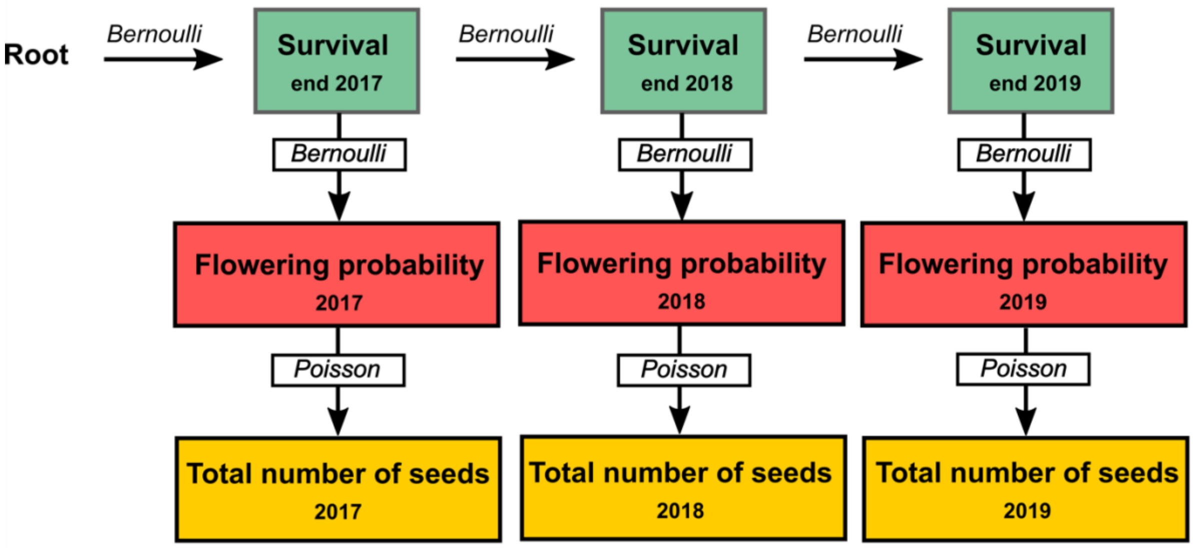

Figure S5. Illustration aster model structure. Aster models were used to predict fitness based on survival, flowering and total seed production. Each node in the figure represents performance in the separate fitness components for each year. Bernoulli and Poisson distribution were used to model survival and flowering probability and total seed production, respectively. Total fitness was combined across years to estimate selection acting on trait values averaged across years (see Material and methods).

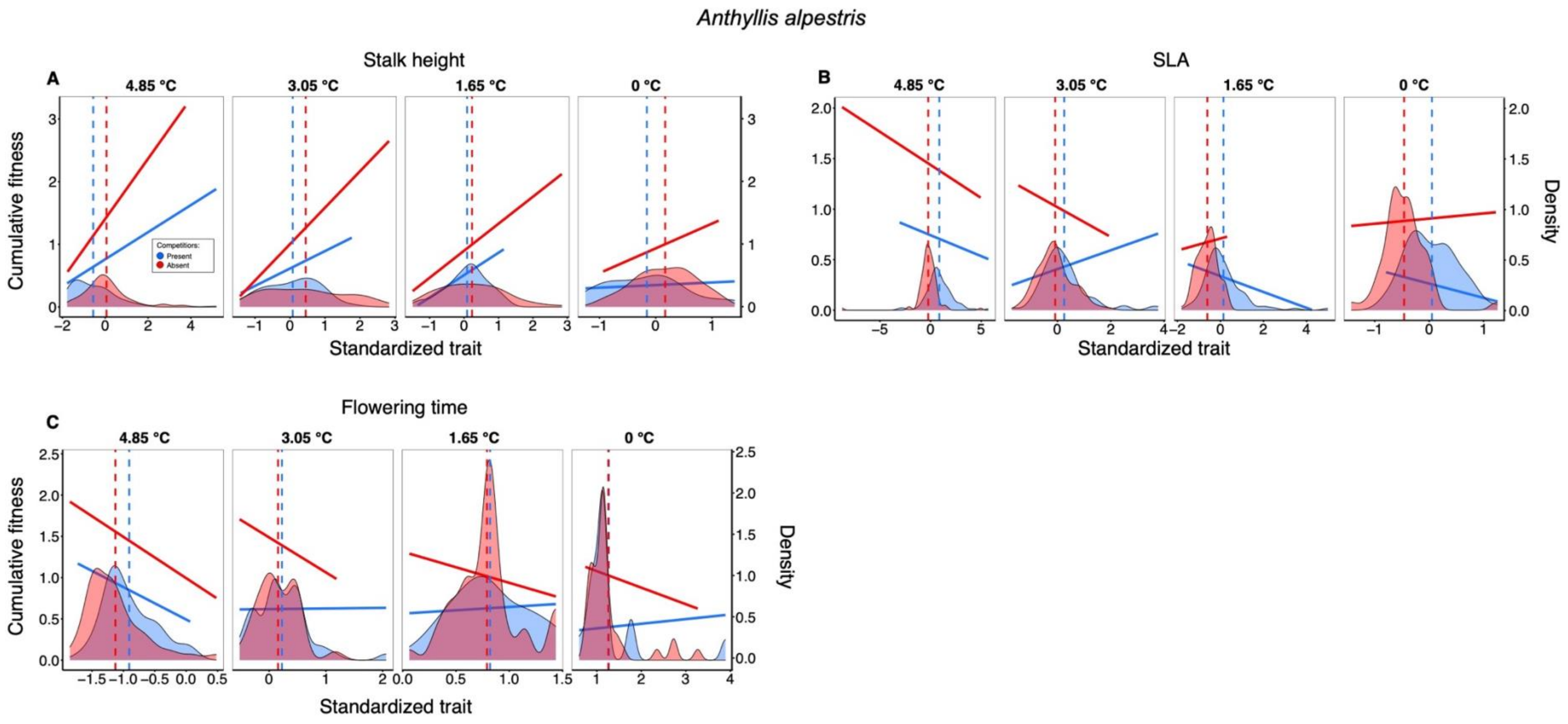

Figure S6. Illustration of selection differentials and trait distribution for stalk height (A), SLA (B) and flowering time (C) across warming levels and competitor treatments for *A. alpestris.* The illustrations of selection differentials (filled lines) are shown for each warming level for the absence (red) and presence (blue) of competitors with standardized trait values on the x-axis and relativized fitness on the y-axis positioned on the left side. Density plots show corresponding trait distribution on the x-axis for

each competitor treatment, with density positioned on the right side of the y-axis. Average trait values are indicated by vertical dashed lines. Illustrations are based on the most parsimonious models for which selection differentials were estimated (Table 1).

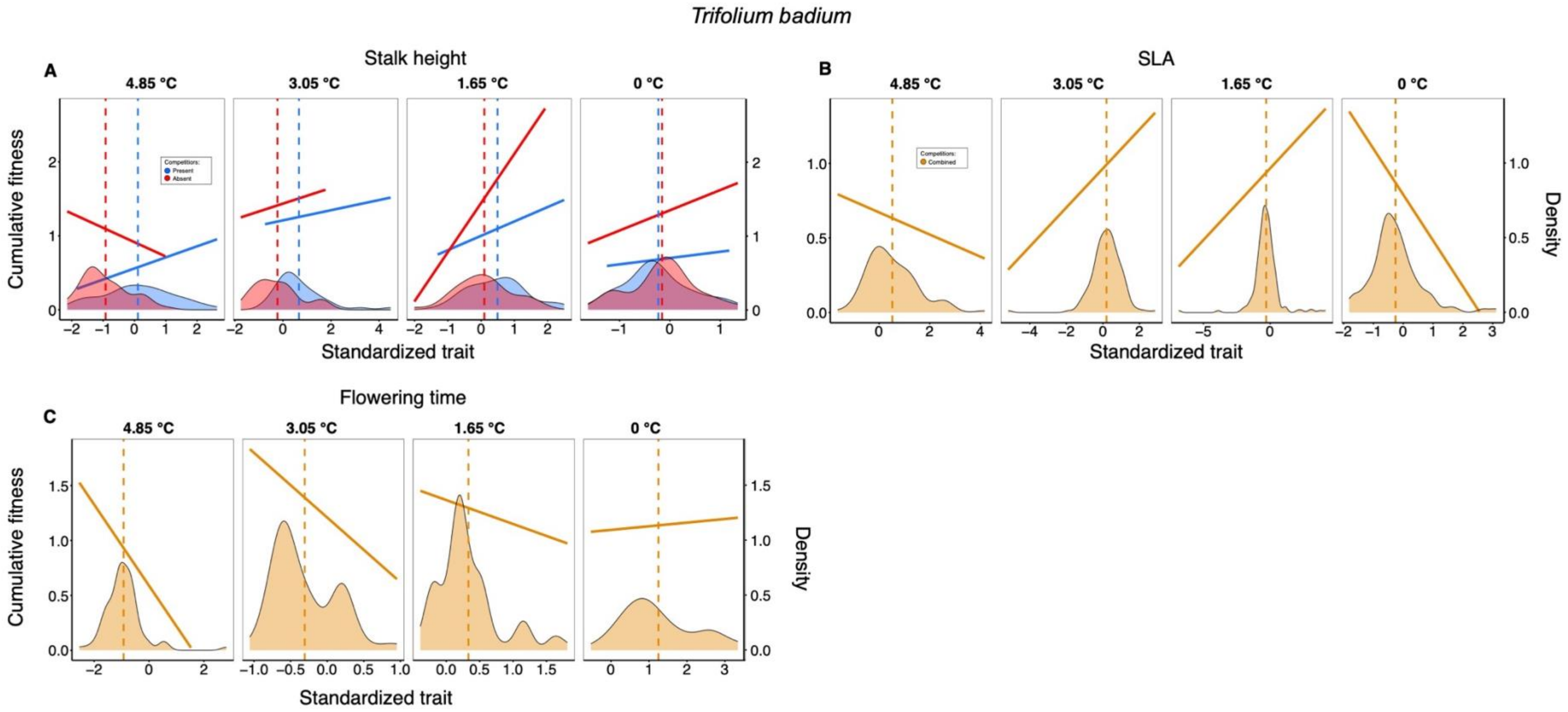

Figure S7. Illustration of selection differentials and trait distribution for stalk height (A), SLA (B) and flowering time (C) across warming levels and competitor treatments for *T. badium.* See figure text Fig. S6.

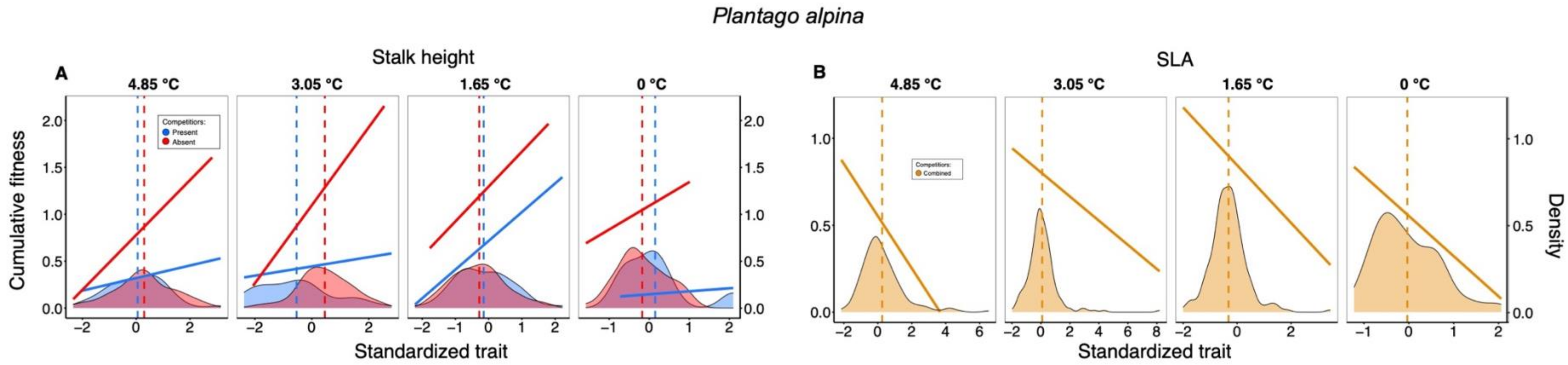

Figure S8. Illustration of selection differentials and trait distribution for stalk height (A) and SLA (B) across warming levels and competitor treatments for *P. alpina.* See figure text Fig. S6.

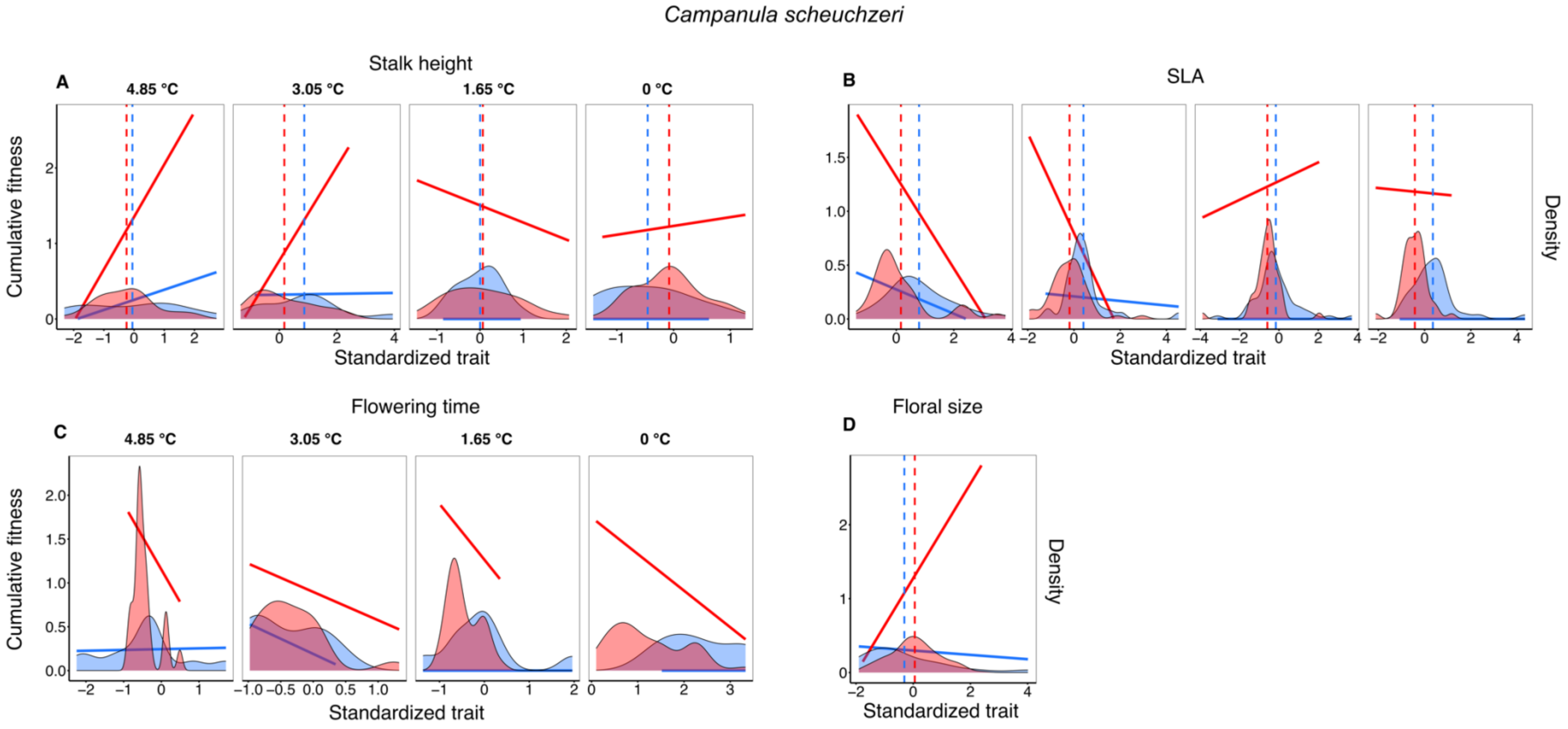

Figure S9. Illustration of selection differentials and trait distribution for stalk height (A), SLA (B), flowering time (C) and floral size (D) across warming levels and competitor treatments for *C. scheuchzeri.* See figure text Fig. S6.

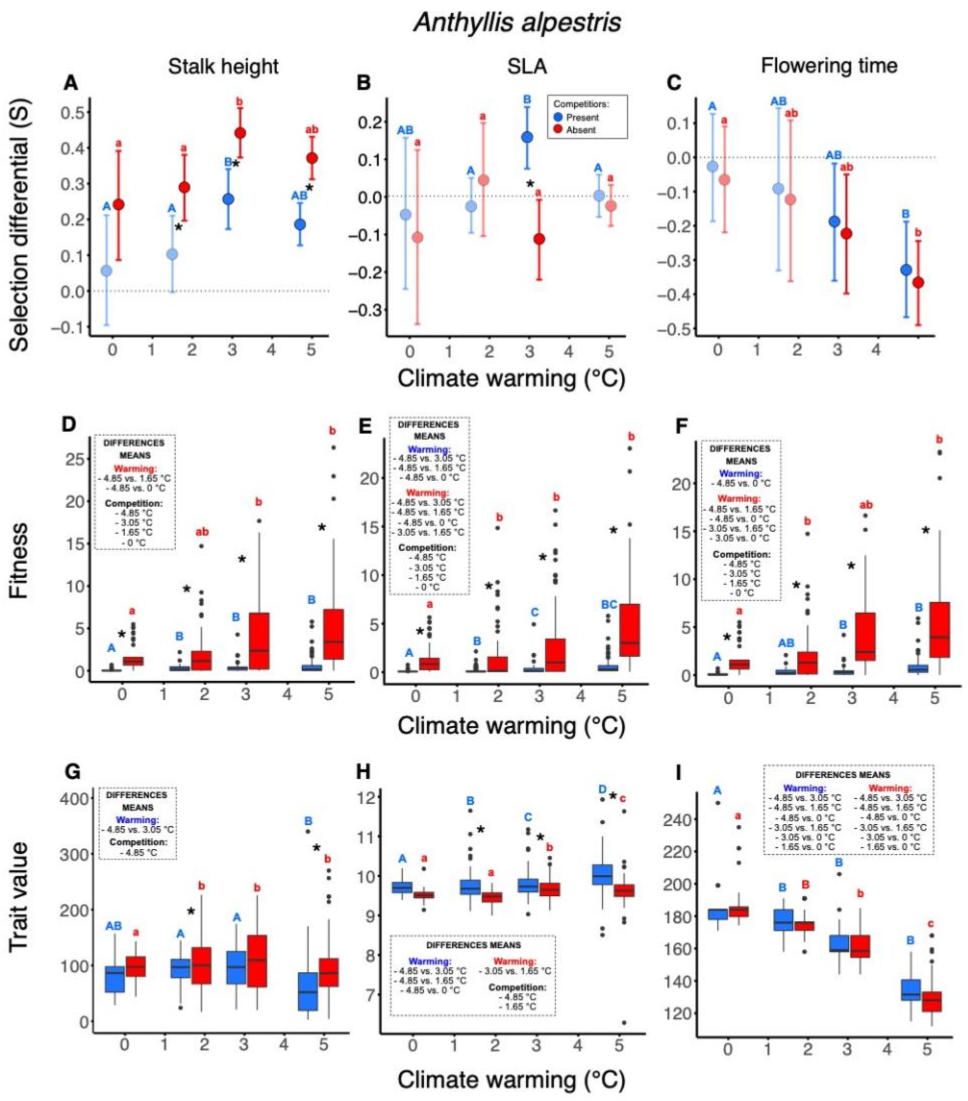

Figure S10. Selection differentials (A-C), the distribution of fitness (D-F) and phenotypic traits (G-I) across factors (warming and competition) for which selection was estimated for *A. alpestris.* The effect of site is plotted as the level of warming on the x-axis, simulated by transplantation to lower elevations (Fig. 1). A-C show estimates and 95% CI from bootstrapped selection differentials (n = 5000) where non-significant selection differentials are plotted as partly transparent. Letters indicate significant differences in selection (A-C), fitness (D-F) and trait variation (G-I) between warming levels when competitors are present (capital letters) or when competitors are absent (lower-case letters). Asterisks indicate significant differences

in selection (A-C), fitness (D-F) and trait variation (G-I) between competitor treatments. Significant differences in absolute means are indicated in insets, where comparisons between warming levels when competitors are present and absent are indicated in blue and red text, respectively.

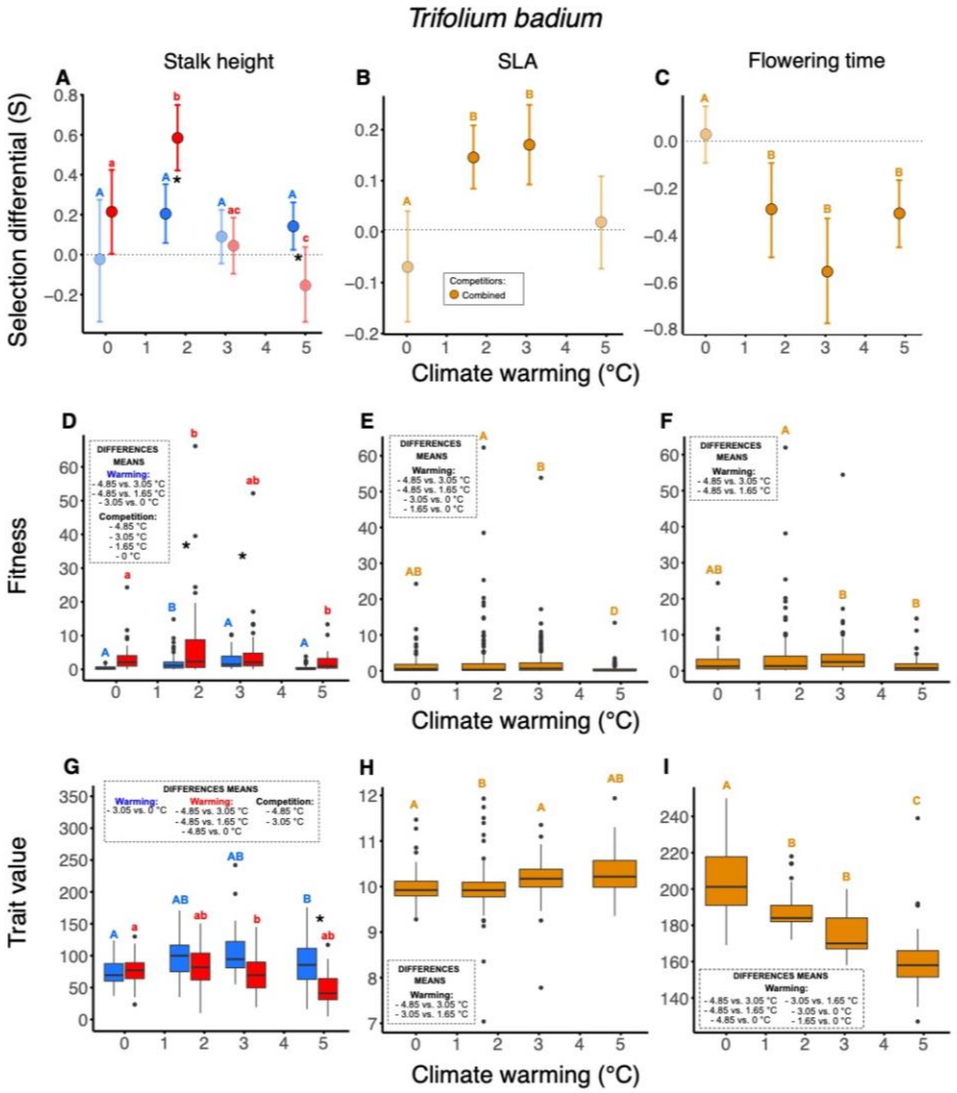

Figure S11. Selection differentials (A-C), the distribution of fitness (D-F) and phenotypic traits (G-I) across factors (warming and competition) for which selection was estimated for *T. badium.* See figure text Fig. S10.

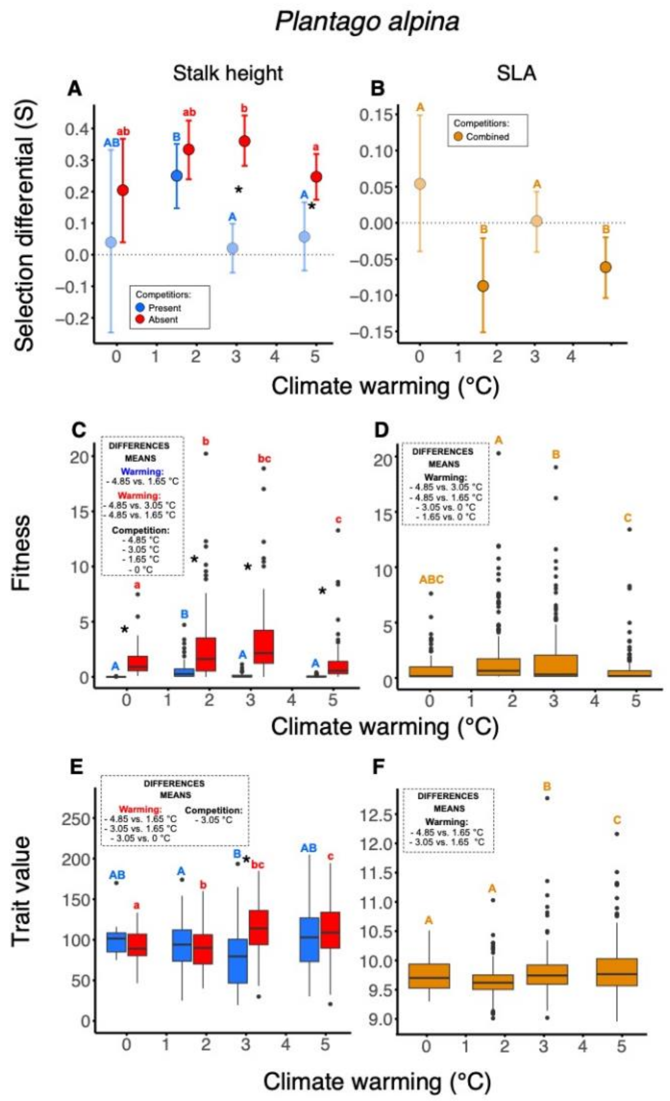

Figure S12. Selection differentials (A-B), the distribution of fitness (C-D) and phenotypic traits (E-F) across factors (warming and competition) for which selection was estimated for *P. alpina.* See figure text Fig. S10.

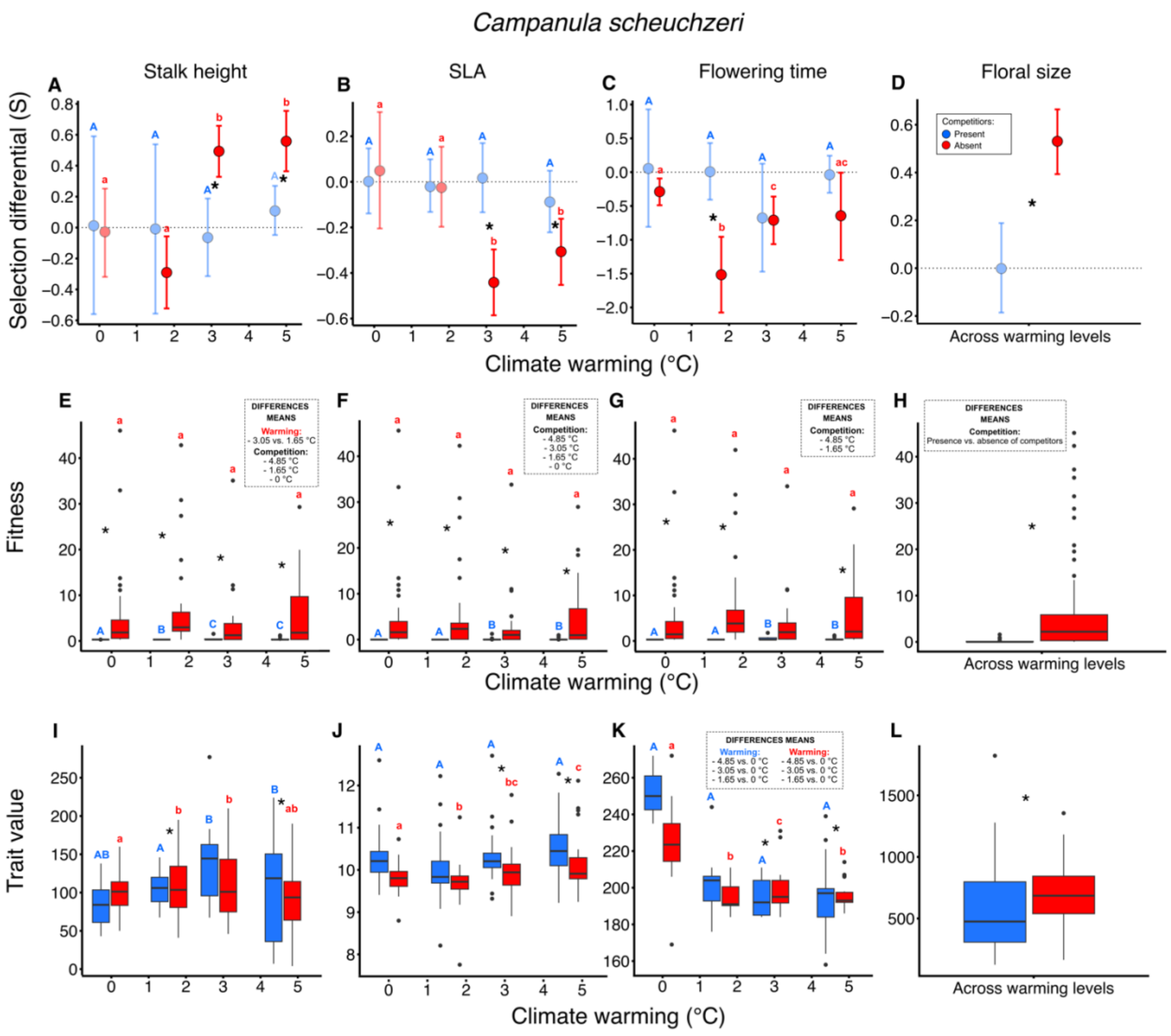

Figure S13. Selection differentials (A-B), the distribution of fitness (C-D) and phenotypic traits (E-F) across factors (warming and competition) for which selection was estimated for *C. scheuchzeri.* See figure text Fig. S10.

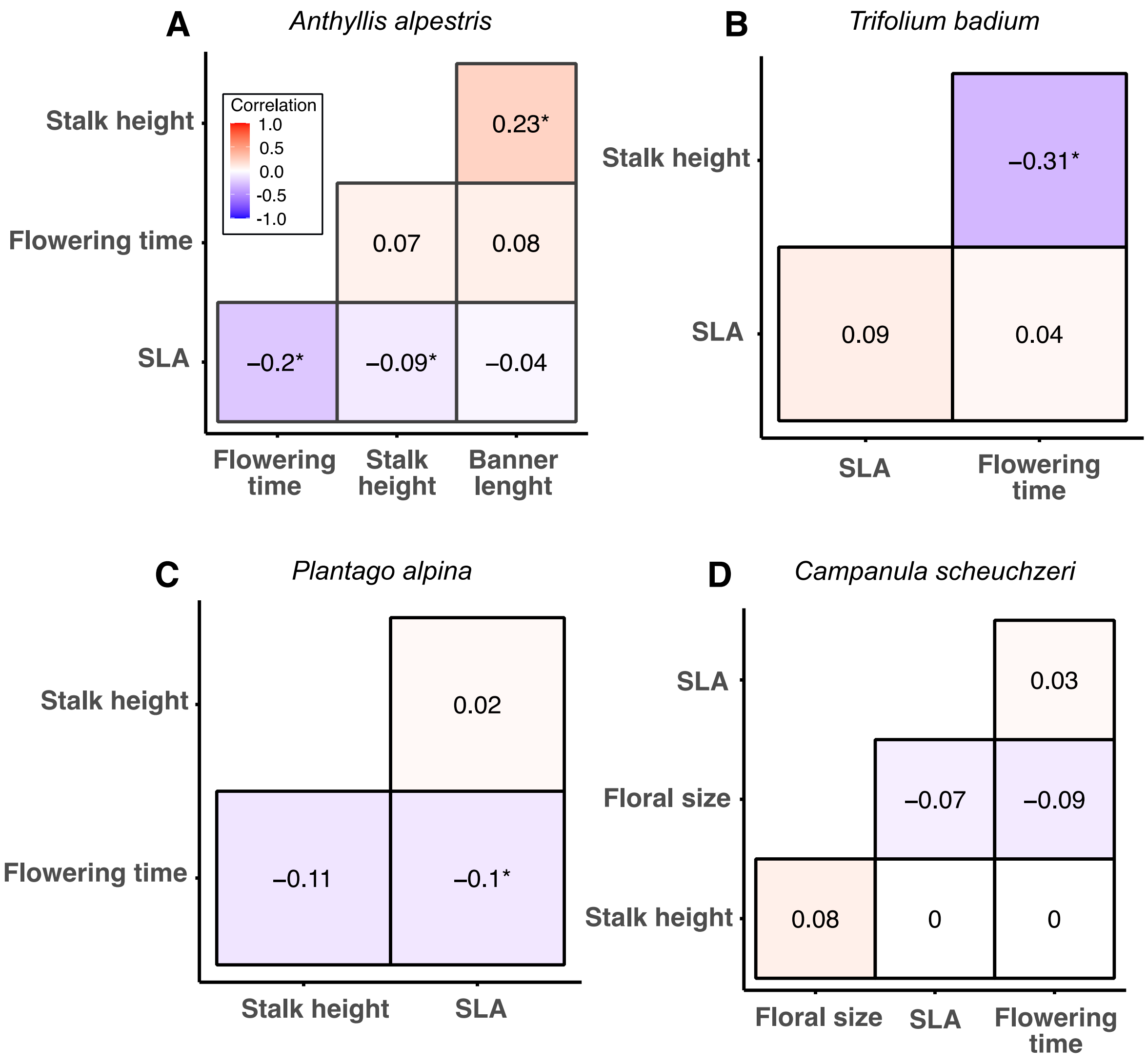

Figure S14. Correlations between traits included in the study across warming levels and competitor treatments for *A. alpestris* (A), *T. badium* (B), *P. alpina* (C) and *C. scheuchzeri* (D). Values show Pearson's correlation coefficient for which significant correlations are indicated by asterisk (*).

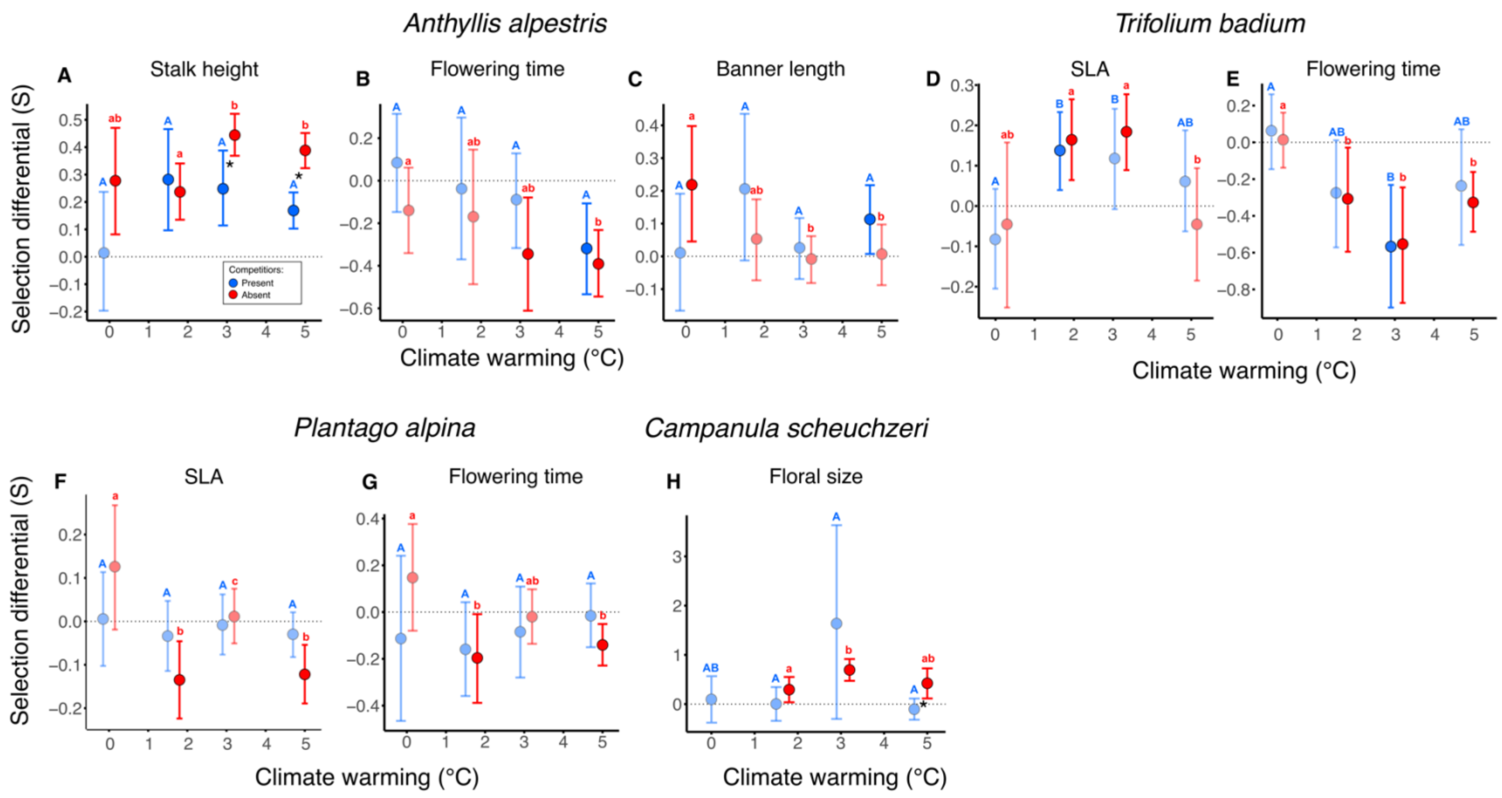

Figure S15. Illustration of selection differentials obtained from full models for *A. alpestris* (A-C), *T.* badium (D-E), *P. alpina* (F-G) and *C. scheuchzeri* (H). The effect of site is plotted as the level of warming on the x-axis, simulated by transplantation to lower elevations (Fig. 1). A-F show estimates and 95% CI from bootstrapped selection differentials (n = 5000) where non-significant selection differentials are plotted as partly transparent. Letters indicate significant differences between warming levels when competitors are present (capital letters) or when competitors are absent (lower-case letters), while asterisks indicate significant

differences between competitor treatments. Only cases for which the most parsimonious models (Table 1) do not equal the full model (Fitness ~ Trait × Site × Competition interaction) are presented here. Note that for selection on floral size in the absence of competitors in the no-warming treatment could not be estimated for *C. scheuchzeri* due to problems with model convergence.

REFERENCES

Athanasiadis, G., Speed, D., Andersen, M.K., Appel, E.V.R., Grarup, N., Brandslund, I. et al. (2020). Estimating narrow-sense heritability using family data from admixed populations. *Heredity*, 124, 751-762.

Andrello, M., De Villemereuil, P., Busson, D., Gaggiotti, O. and Till-Bottraud, I. (2016). Population dynamics of Arabis alpina in the French Alps: evidence for demographic compensation. *bioRxiv*, 070847.

Catchen, J., Hohenlohe, P.A., Bassham, S., Amores, A. and Cresko, W.A. (2013). Stacks: an analysis tool set for population genomics. *Molecular Ecology*, 22, 3124-3140.

Chen, I.-C., Hill, J.K., Ohlemüller, R., Roy, D.B., Thomas, C.D. (2011). Rapid Range Shifts of Species Associated with High Levels of Climate Warming. *Science (New York, N.Y.)*, 333, 1024-1026.

Chesshire, P.R., McCabe, L.M. and Cobb, N.S. (2021). Variation in Plantandndash;Pollinator Network Structure along the Elevational Gradient of the San Francisco Peaks, Arizona. *Insects*, 12, 1060.

Chong, Z., Ruan, J. and Wu, C.-I. (2012). Rainbow: an integrated tool for efficient clustering and assembling RAD-seq reads. *Bioinformatics*, 28, 2732-2737.

Fu, L., Niu, B., Zhu, Z., Wu, S. and Li, W. (2012). CD-HIT: accelerated for clustering the next-generation sequencing data. *Bioinformatics*, 28, 3150-3152.

Garrison, E., and G. Marth. (2012). Haplotype-based variant detection from short-read sequencing. *arXiv preprint* arXiv:1207.3907.

Hanzawa, F.M. and Kalisz, S. (1993). The Relationship between Age, Size, and Reproduction in Trilliu grandiflorum (Liliaceae). *Am J Bot*, 80, 405-410.

Kirkpatrick, M. (1984). Demographic Models Based on Size, Not Age, For Organisms with Indeterminate Growth. *Ecology*, 65, 1874-1884.

Körner, C. (2003). *Alpine plant life functional plant ecology of high mountain ecosystems*. 2nd edn. Springer, Berlin.

Lande, R. and Arnold, S.J. (1983). The Measurement of Selection on Correlated Characters. *Evolution*, 37, 1210-1226.

Lauenroth, W.K. and Adler, P.B. (2008). Demography of perennial grassland plants: survival, life expectancy and life span. *Journal of Ecology*, 96, 1023-1032.

Li, H. and Durbin, R. (2009). Fast and accurate short read alignment with Burrows–Wheeler transform. *Bioinformatics*, 25, 1754-1760.

Menges, E.S. (2000). Population viability analyses in plants: challenges and opportunities. *Trends Ecol Evol*, 15, 51-56.

National Centre for Climate Services (2018). CH2018 – Climate Scenarios for Switzerland, Technical Report. National Centre for Climate Services, Zurich, 271 pp. ISBN: 978-3-9525031-4-0

Nomoto, H.A. and Alexander, J.M. (2021). Drivers of local extinction risk in alpine plants under warming climate. *Ecology Letters*, 24, 1157-1166.

Ollerton, J. and Lack, A. (1998). Relationships between flowering phenology, plant size and reproductive success in shape Lotus corniculatus (Fabaceae). *Plant Ecology*, 139, 35-47.

Parmesan, C. (2006). Ecological and Evolutionary Responses to Recent Climate Change. *Annual Review of Ecology, Evolution, and Systematics*, 37, 637-669.

Perkins, L.D., Parks, G.C., Dwire, K.A., Endress, B.A. and Kelsi, L.J. (2006). Age Structure and Age-Related Performance of Sulfur Cinquefoil (Potentilla recta). *Weed Science*, 54, 87-93

Peterson, B.K., Weber, J.N., Kay, E.H., Fisher, H.S. and Hoekstra, H.E. (2012). Double digest RADseq: an inexpensive method for de novo SNP discovery and genotyping in model and non-model species. *PLoS One*, 7, e37135.

Pop, M. and Salzberg, S.L. (2008). Bioinformatics challenges of new sequencing technology. *Trends Genet*, 24, 142-149.

Puritz, J.B., Hollenbeck, C.M. and Gold, J.R. (2014). dDocent: a RADseq, variant-calling pipeline designed for population genomics of non-model organisms. *PeerJ*, 2, e431-e431.

Richman, S.K., Levine, J.M., Stefan, L., Johnson, C.A. (2020). Asynchronous range shifts drive alpine plant–pollinator interactions and reduce plant fitness. *Global Change Biology*, 26, 3052-3064.

Rowe, A.K., Rutledge, S.A., Lang, T.J., Ciesielski, P.E. and Saleeby, S.M. (2008). Elevation-Dependent Trends in Precipitation Observed during NAME. *Monthly Weather Review*, 136, 4962-4979.

Troth, A., Puzey, J.R., Kim, R.S., Willis, J.H. and Kelly, J.K. (2018). Selective trade-offs maintain alleles underpinning complex trait variation in plants. *Science* (New York, N.Y.), 361, 475-478.

Valencia, E., Méndez, M., Saavedra, N. and Maestre, F.T. (2016). Plant size and leaf area influence phenological and reproductive responses to warming in semiarid Mediterranean species. *Perspectives in plant ecology, evolution and systematics*, 21, 31-40.

Van Beusekom, A.E., González, G. and Rivera, M.M. (2015). Short-Term Precipitation and Temperature Trends along an Elevation Gradient in Northeastern Puerto Rico. *Earth Interactions*, 19, 1-33.

Westergaard, K.B., Zemp, N., Bruederle, L.P., Stenøien, H.K., Widmer, A. and Fior, S. (2019). Population genomic evidence for plant glacial survival in Scandinavia. Mol Ecol, 28, 818-832.
